# Electronic Nose for Improved Environmental Methane Monitoring

**DOI:** 10.1021/acs.est.3c06945

**Published:** 2023-12-21

**Authors:** Guillem Domènech-Gil, Nguyen Thanh Duc, J. Jacob Wikner, Jens Eriksson, Sören Nilsson Påledal, Donatella Puglisi, David Bastviken

**Affiliations:** † Department of Thematic Studies and Environmental Change (TEMAM), Linköping University, Linköping 58183, Sweden; ‡ Department of Electrical Engineering (ISY), Linköping University, Linköping 58183, Sweden; § Department of Physics, Chemistry, and Biology (IFM), Linköping University, Linköping 58183, Sweden; ∥ Tekniska Verken i Linköping AB, Box 1500, Linköping 581 15, Sweden

**Keywords:** greenhouse gas, machine learning, gas sensors, low-cost

## Abstract

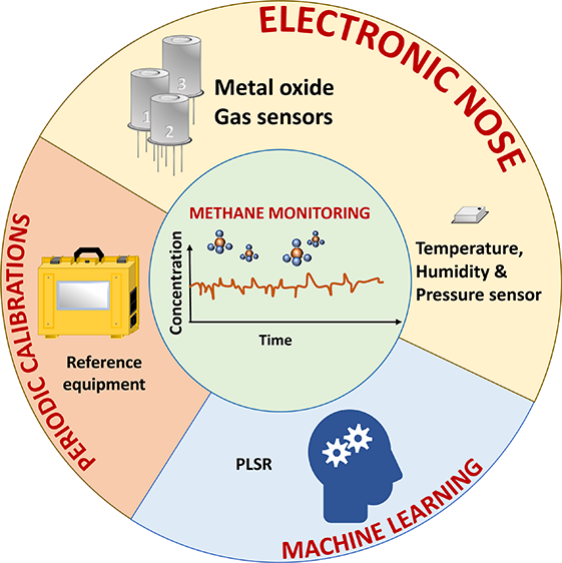

Reducing emissions of the key greenhouse gas methane (CH_4_) is increasingly highlighted as being important to mitigate climate change. Effective emission reductions require cost-effective ways to measure CH_4_ to detect sources and verify that mitigation efforts work. We present here a novel approach to measure methane at atmospheric concentrations by means of a low-cost electronic nose strategy where the readings of a few sensors are combined, leading to errors down to 33 ppb and coefficients of determination, *R*^2^, up to 0.91 for in situ measurements. Data from methane, temperature, humidity, and atmospheric pressure sensors were used in customized machine learning models to account for environmental cross-effects and quantify methane in the ppm–ppb range both in indoor and outdoor conditions. The electronic nose strategy was confirmed to be versatile with improved accuracy when more reference data were supplied to the quantification model. Our results pave the way toward the use of networks of low-cost sensor systems for the monitoring of greenhouse gases.

## Abstract 2

Environmental methane tracking techniques at affordable prices need to be established. Our work enables methane monitoring in most types of environments with low-cost sensing technologies.

## 1 Introduction

Atmospheric methane (CH_4_) concentrations have been increasing rapidly since preindustrial times, from about 0.7 to more than 1.8 parts per million (ppm).^1^ The CH_4_ global warming potential per mass on a 100 year time horizon is 28–34 times greater than for carbon dioxide.^2^ Furthermore, the CH_4_ increase has been irregular for unknown reasons, meaning that emission sources and sinks along with flux magnitudes and regulation are in many cases poorly understood.^3^ Hence, better ways to monitor CH_4_ at local scales are essential to reveal source-sink dynamics in time and space and to identify where and when mitigation efforts are needed and to validate their efficiency. Low-cost sensors have been proposed as a useful solution for rendering certain types of CH_4_ monitoring more affordable,^4,5^ thereby supplementing other methods, such as satellite surveillance, aircraft sampling, or ground-based micrometeorological measurements.^6,7^ However, important aspects remain to be investigated to assess the potential of low-cost sensors. A particular bottleneck is the lack of versatile systematic calibration and interference correction, which limits the performance and sensitivity of the measurements and restricts the types of low-cost sensor applications that are considered reliable.^8^ While laboratory calibrations with only CH_4_ as the gas giving sensor response yield “clean” calibration curves, outdoor use suffers from large interferences from, e.g., water vapor (H_2_O(g)), and multidimensional calibration is needed for reliable use in outdoor environments.^5^ It has also been challenging to reach high-performance CH_4_ measurements (e.g., resolving small changes around concentrations as low as the ambient atmospheric CH_4_ concentrations) under variable H_2_O(g) concentrations with single sensors in spite of advanced data processing approaches including machine learning.^4,9^ Here, we approach this challenge by a combination of multivariable calibration and using an electronic nose (e-nose) approach with integrated multivariate analysis of simultaneous data from multiple sensors in low-cost sensor systems (LCSSs) designed to monitor CH_4_ concentrations.

An e-nose is an electronic system formed by an array of gas sensors. Each of the sensors constituting the e-nose is supposed to report a different response pattern to the gas or gas mixture to be measured. The aim of this is to better resolve and quantify the gases present in a mixture and their concentrations by combining many different sensor readings in the data treatment.^10^ We used Arduino-controlled Figaro TGS2600-family CH_4_ sensors, operating at 16-bit resolution, and developed CH_4_ calibration routines to manage the strong interactions of H_2_O(g) and other parameters outdoors such as temperature (*T*) and barometric pressure (*P*). This enabled extraction of information about CH_4_ concentrations observed in our measurement campaigns, ranging from 1 to 150 ppm. The aims included developing and evaluating the e-nose-LCSS approach to open for more reliable low-cost alternatives in various types of environmental monitoring of greenhouse gas (GHG) emissions.

## 2 Materials and Methods

### 2.1 Sensors

We used TGS2611-C00 (TGSC)^11^ and TGS2611-E00 (TGSE)^12^ sensors from Figaro Engineering Inc. (Illinois, USA) to measure CH_4_. These two types of sensors are based on the same metal oxide material (SnO_2_) that, when heated, shows sensitivity to CH_4_. The main difference among these sensors is that TGSE is equipped with a filter that reduces the cross-sensitivity to other combustible gases such as hydrogen gas, non-CH_4_ alkanes, and alcohols, making it more selective toward CH_4_. They are both commercially available at affordable prices (∼€30 each; year 2022) and with a compact and robust design, packaged in the electronics standard design TO-5 to facilitate their integration with the gas measurement equipment. These sensors show a power consumption of about 300 mW, from which 15 mW is used to measure the conductivity changes of the sensing material and about 280 mW for the heater. The factory calibration is performed between 300 and 10,000 ppm, and their main intended application is leakage detection. The response time of these sensors is below 30 s for 5000 ppm of CH_4_ in dry air.

The sensor used to measure relative humidity (RH), *T*, and *P* was BME680 from Bosch Sensortec GmbH (Reutlingen, Germany).^13^ BME680 covers measurement ranges from 0 to 100% RH with an accuracy of ±3%, −40 to 85 °C with an accuracy of ±1 °C, and 300 to 1100 hPa with an absolute accuracy of ±1 hPa. According to the manufacturer, BME680 unsoldered offers a response time (τ_33–63%_) of 0.75 s. This sensor, working in continuous mode, has a power consumption of about 40 mW.

During the measurements in the laboratory, a digital humidity sensor, SHTC1 from Sensirion AG (Switzerland),^14^ was used to monitor the RH and *T*. The SHTC1 sensor covers a measurement range from 0 to 100% RH with a typical accuracy of ±3% and from −30 to 100 °C with a typical accuracy of ±0.3 °C. This sensor has an average power consumption of 8.6 μW. SHTC1 was used in the laboratory instead of BME680 because this sensor was already integrated with the equipment and software.

### 2.2 Reference Equipment

A custom-built gas mixing system (GMS) equipped with six digital Mass-Flow Controllers (MFCs) from Bronkhorst High-Tech (Ruurlo, the Netherlands) commanded via *C* software, delivering gas mixtures at ambient *P*, and connected to a computer via serial communication was used as in-lab reference equipment. The MFCs were previously calibrated by Bronkhorst. This equipment allowed us to supply different gas mixtures and was used for laboratory calibrations.

As field measurement reference instruments, we used Greenhouse Gas Analyzers (GGAs) by Los Gatos Research, an ultraportable 915-011 (UGGA) or a DLT100 for CH_4_, CO_2_, and H_2_O(g),^15^ both with an operational range from 0 to 500 ppm of CH_4_ and an accuracy of 2 ppb.

The GMS and the UGGA were calibrated with an Agilent 7890A gas chromatograph (GC) system with a flame ionization detector coupled to a 7697A Headspace sampler verified with certified gas standards. Uncertainties relative to GC were below 3% (Figure S1) and below 5.5% for GMS and UGGA, respectively.

### 2.3 Sensor Characterization

The TGS sensors were calibrated with the GMS inside a custom-made stainless-steel chamber of about 400 mL of volume by exposing them to different concentrations of CH_4_, ranging from 1 to 9 ppm, diluted in humid synthetic air (SA) with H_2_O(g) concentrations ranging from 4.5 to 14.0 g·m^–3^. The ranges of concentrations studied were chosen to reproduce situations commonly faced under in situ field conditions. H_2_O(g) was controlled by letting part of the SA flow through a bubbler. The gas flow corresponding to each H_2_O(g) concentration was calibrated with the SHTC1 sensor at room temperature (20 °C) before characterization of the sensors. For all the gas measurements, a total constant flow of 100 mL/min was kept. The gas measurements include (i) allowing the device to stabilize the baseline for four h and (ii) exposing the devices for one h to different concentrations of CH_4_ at each H_2_O(g) level. No relaxation time or air flushing between CH_4_ concentration changes was included to simulate, as much as possible, real in situ situations where the concentrations of gases increase or decrease from an already existing concentration. We used the LCSS platforms (described below) to acquire and record the sensor signals for sensor characterization with the GMS.

To evaluate conditions inside the gas chamber when exposed to active sensors releasing heat, *T* was measured at three locations before starting the calibration: ingoing gas immediately before entering the chamber and two locations inside the chamber (near the inlet and in the central part of the chamber). Although *T* and RH varied, H_2_O(g) was stable along the chamber (Table S1). After verifying homogeneous conditions, the SHTC1 sensor was used in the central part of the chamber during the calibration measurements.

Each sensor has a different baseline and responds differently to CH_4_ and influencing parameters such as H_2_O(g), *T*, and *P* due to structural details or positioning of the sensing material affecting the kinetic energy of the impinging gas molecules. For this reason, each sensor was assigned a unique polyethylene sticker label (KA Etikettering Sverige AB, Sweden) allowing for sensor-specific calibration and data tracking. Measurements with the bare sensors and then the same sensors with the labels, respectively, confirmed that the stickers used did not influence the measurements (Figure S2).

### 2.4 Sensor Systems

**Figure 1:**
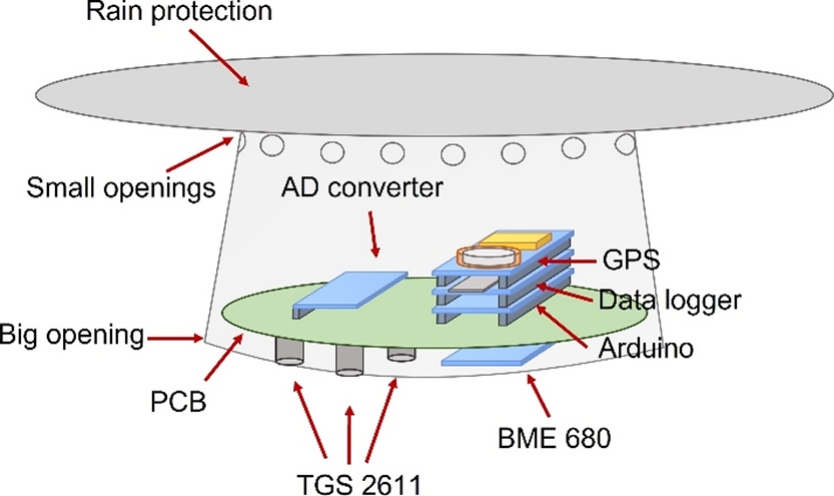
Schematics of the LCSS equipped with an analog-to-digital converter, a global positioning system, a data logger, an Arduino microcontroller, a BME680 sensor, two TGS2611-E00 sensors, one TGS2611-C00 sensor, and the housing used for field measurements.

Laboratory calibrations with gas mixtures at different H_2_O(g) levels were supplemented by field tests where the LCSSs were tested in situ toward CH_4_ levels and other influencing parameters (mainly H_2_O(g), *T*, *P*, and possible interfering gases). The LCSS consisted of a tailor-made printed circuit board (PCB) that in this case powered and controlled three TGS sensors (one TGSC and two TGSE) and one BME680 sensor via an Arduino MKR WAN 1310 from Arduino AG (Mainz, Germany), a 16-bit analog-to-digital converter ADS1115 from Adafruit Industries (New York, USA), and an in-house *C* code uploaded using the Visual Studio software and Platform IO extension. The data acquired by the different sensors was logged to a secure digital card (SD card) on an MKR SD Proto Shield from Arduino AG together with the time, date, and geolocalization reported by the global positioning system (GPS; by an Arduino MKR GPS Shield from Arduino AG) at 1 min intervals. The LCSSs were powered with 12 or 9 V transformers. For field measurements, the LCSSs were protected from rain with a housing, made from modified low-cost PE lunch jars and cutting boards, in which the sensors were directly exposed to air through a big bottom opening, while the design promoted convective air movement across the system by several small top openings, as illustrated in Figure 1.

Although the sensors in the LCSS were continuously powered, allowing 1 Hz readings, only one data block per minute was logged on the SD card. This was done to reduce the log file sizes when measuring for long periods and as a compromise to simultaneous wireless data transfer tests (outside of the scope of this study). GPS data were used to assign each data block with a date, time, latitude, longitude, and altitude. For every TGS sensor in the LCSS, two values per minute were logged in the SD card: the mean and standard deviation, which were calculated from 10 readings (one every 6 s) along 1 min. The BME680 data logged in the SD card were three values per minute (RH, *T*, and *P*) from the reading right before data storage.

### 2.5 Field Tests

Different environmental conditions were chosen to test the sensor quantification models in various in situ applications. The field sites represent a variety of indoor and outdoor cases that included different concentrations and ranges of CH_4_, H_2_O(g), *T*, and *P*, possibly different interfering gases, and with CH_4_ emissions arising from either anthropogenic or natural sources. Table 1 summarizes the mean and standard deviation of the selected variables recorded at each field site. The chosen sites, all located in Sweden, were (1) outdoors in a private garden in a suburban area close to a forest during autumn; (2) near sludge piles stored outdoors at a municipal wastewater treatment plant from spring to summer; (3) inside a wastewater sludge pressing screw room (facility of the wastewater treatment plant with pressing screw devices to dewater sludge); and (4) in a hemiboreal wetland during late summer. The H_2_O(g) concentrations measured in the field sites were within the range studied in the laboratory (4.5–14.0 g·m^–3^).

**Table 1:** In Situ Measurement Sites Used in This Study^a^

site	RH (%)	*T* (°C)	*P* (hPa)	H_2_O(g) (g·m^–3^)	CH_4_ (ppm)
1. garden	63.4 ± 9.8	9.9 ± 3.2	1008.0 ± 8.8	5.9 ± 0.6	1.97 ± 0.04
2. sludge piles	54.1 ± 15.5	17.4 ± 7.4	1011.9 ± 10.2	8.0 ± 1.2	2.68 ± 0.99
3. sludge screw room	23.9 ± 8.4	30.1 ± 2.8	1011.4 ± 11.0	7.3 ± 0.5	33.8 ± 27.2
4. wetland	56.6 ± 10.9	23.4 ± 3.8	1008.6 ± 2.9	11.9 ± 0.7	2.42 ± 1.06

a Notes: mean values and standard deviations of the relative humidity, temperature, barometric pressure, water vapor, and methane concentrations for the measurement periods at each site are provided.

Several LCSS instruments were deployed at each field site and were allowed to measure continuously. Reference measurements used to train the quantification model were generated in two different ways: (i) continuously for multiple days and (ii) repeatedly during shorter periods (30 min) at different times of the day or night to cover different environmental conditions. For these measurements, the GGA inlet was placed within 5 cm of the TGS sensors. DLT100 was used as a reference instrument for the garden site, and the UGGA was used for the other locations requiring greater mobility. Because most of the measurements were performed in parallel after the laboratory calibrations, each sensor was tested in the laboratory and then used at one or two of the field sites.

### 2.6 Data Evaluation

To study and quantify the individual and interaction effects of CH_4_ and H_2_O(g) on the signal of each type of TGS sensors, all the data obtained from the calibrations done with the GMS were used to perform a general linear model (GLM) with two variables,^16^ CH_4_ and H_2_O(g).

To approach the CH_4_ quantification with an e-nose strategy, partial least-squares regression (PLSR)^17^ models were trained and tested using either the data extracted from all three TGS sensors in an LCSS and the SHTC1 or from all three TGS sensors and the BME680 in an LCSS. PLSR is a machine learning technique based on multivariate statistics that is able to find relations between a matrix of predictors (*X*) and a matrix of responses (*Y*), and it is a well-suited technique for dealing with multicollinearity. PLSR finds the direction in *X* that explains the maximum variance direction in *Y* and returns the coefficients of a multivariable linear regression. *X* has *n* × *m* dimensions, where *n* is the number of observations and m is the number of chosen predictor variables (or features). In our case, the rows of data acquired with one LCSS (one data record per minute) represent the observations, whereas the columns represent the features. The columns in our data files correspond to the signal of a certain sensor or information derived from it (as explained in the Supporting Information). *Y* has dimensions of *n* × *p*, where *n* is again the number of observations and *p* is the number of responses. In our case, the responses in the *Y* matrix are each row of data acquired with reference equipment (GMS and GGAs). Once the PLSR finds the direction in *X* that explains the direction of maximum variance in *Y*, a selected number of observations of the features is used to obtain predictions of the response values. To evaluate the performance of the PLSR model, these predictions are compared to values reported by the reference equipment (GMS or GGA data) that were not used to calculate the regression coefficients.

For the laboratory measurements, the observations (*n*) constituting the rows of the predictor matrix (*X*) are not all of the data acquired with the LCSS but a selection of it. The selected observations are certain regions of interest (ROIs, Figure S3) in the measurements. These ROIs correspond to the moments when the TGS sensors reached a steady state once exposed to the different CH_4_–H_2_O(g) combination studied. The steady states were periods of 40 min corresponding to 10 min after and 10 min before every change of CH_4_ concentration. Each ROI is equivalent to 40 data points. Because the sensors were exposed to seven concentrations of CH_4_ in the laboratory (0, 1.0, 1.5, 2.0, 2.5, 5.0, and 9.0 ppm) and each of them under seven concentrations of H_2_O(g) (4.5, 5.2, 6.9, 8.6, 10.4, 12.1, and 14.0 g·m^–3^), a total of 1960 observations were collected per measurement. The data from the three TGS sensors in each LCSS were selected along with the mean H_2_O(g) concentration measured with SHTC1 as features. Thus, for the laboratory measurements, we obtained a predictor matrix (*X*) of 1960 rows (*n* observations) and four columns (*m* features) and a response matrix (*Y*) of 1960 rows and one column (*p* responses).

When using the raw signals of the sensors as features for the field measurements, PLSR generated predictions with poor correlation with the reference data (0.04 < *R*^2^ < 0.53). In certain occasions, even negative concentrations were obtained as predictions. To overcome this issue, we extracted additional information from the signal of the sensors (referred to as “secondary (data) features”). Thus, secondary features extracted from the signal of the TGS sensors are the following three: mean value, slope, and fast Fourier transform (details in the Supporting Information). Because the data from the sensors were logged every minute and we needed to use at least two data points to derive any new secondary features, 2 min intervals were used to calculate such features. Due to these mathematical transformations involving 2 min intervals, we obtained half of the initial observations for the TGS sensors and misalignments with the reference data. To align the observations of the LCSS with the observations of the reference equipment, mean values of 2 min intervals were calculated also for the data from the reference equipment. Following this procedure, we obtained three features from each of the three TGS sensors and three features from the BME680 (mean value of RH, *T*, and *P*), resulting in a total of 12 features or columns of the predictors matrix (*X*). The response variable of the response matrix (*Y*) was the two min mean values of the reference CH_4_ concentrations acquired with the GGAs. The number of observations for both matrices depends on the number of measurements performed with the reference equipment in each field site studied. The same model (or algorithm) was trained separately with the data of each field site to obtain the regression coefficients for each case, and data from the field sites and laboratory measurements were not merged.

Before training the PLSR regression model, the features and *Y* matrix were *Z*-standardized (by subtracting the mean value of all of the data points to each data point and dividing the result by the corresponding standard deviation). Standardization is necessary when using PLSR to enable an equal contribution from the different variables studied while ensuring that variables with larger scales do not dominate, which would induce biased and unreliable results. Consequently, once the model is trained and we want to test it, the model output variables need to be back standardized (by multiplying by the standard deviation of the training data set and adding the corresponding mean value) to obtain meaningful interpretations of accuracy and to evaluate the performance of the PLSR regression model on the original scale of the data.

The reliability and error estimation robustness of the PLSR models elaborated here were improved by preparing them with a 10-fold cross-validation^18^ as follows: (i) the data (observations) were randomly split in ten smaller sample sets, i.e., ten data subsets that contain 10% of the total data set and (ii) eight out of the ten data subsets (80% of the data) were randomly selected and Z-standardized and used for training the model obtaining a set of relations that allow us to transform the predictors into the response. The remaining two data subsets (20% of the data) were Z-standardized and reserved for testing the quantification relations obtained with the training data by transforming the nonused *X* data to predicted *Y* data (CH_4_ concentrations) and comparing it with the observed *Y* data (reference data from GMS or UGGA in our case) by means of different measures of error—the coefficient of determination (*R*^2^)^19^ and the root-mean-square error (RMSE)^16,18^; (iii) the procedure of (ii) was repeated ten times, each time reserving two different data subsets nonpreviously used together for testing while saving the results of the different measures of error of every iteration; (iv) all the *R*^2^ and RMSE results from the ten iterations were averaged and root-mean-square averaged, respectively, to generate a global *R*^2^ and RMSE as superior measures of error and performance of the model than if we would train the model just once and with 100% of the data. In this way, and taking into consideration that the training process of PLSR already accounts for error minimization, the performance of the model obtained is likely to be representative of the data.

Overfitting, which would give a model that only corresponds to a particular set of data and, therefore, would fail outside the particular data domain studied, is always a risk in complex data treatment. We tried to minimize this risk by performing cross-validation and maintaining the number of observations (rows) in the X matrix always being much higher than the number of features (columns).

## 3 Results and Discussion

### 3.1 Sensor Measurements

**Figure 2:**
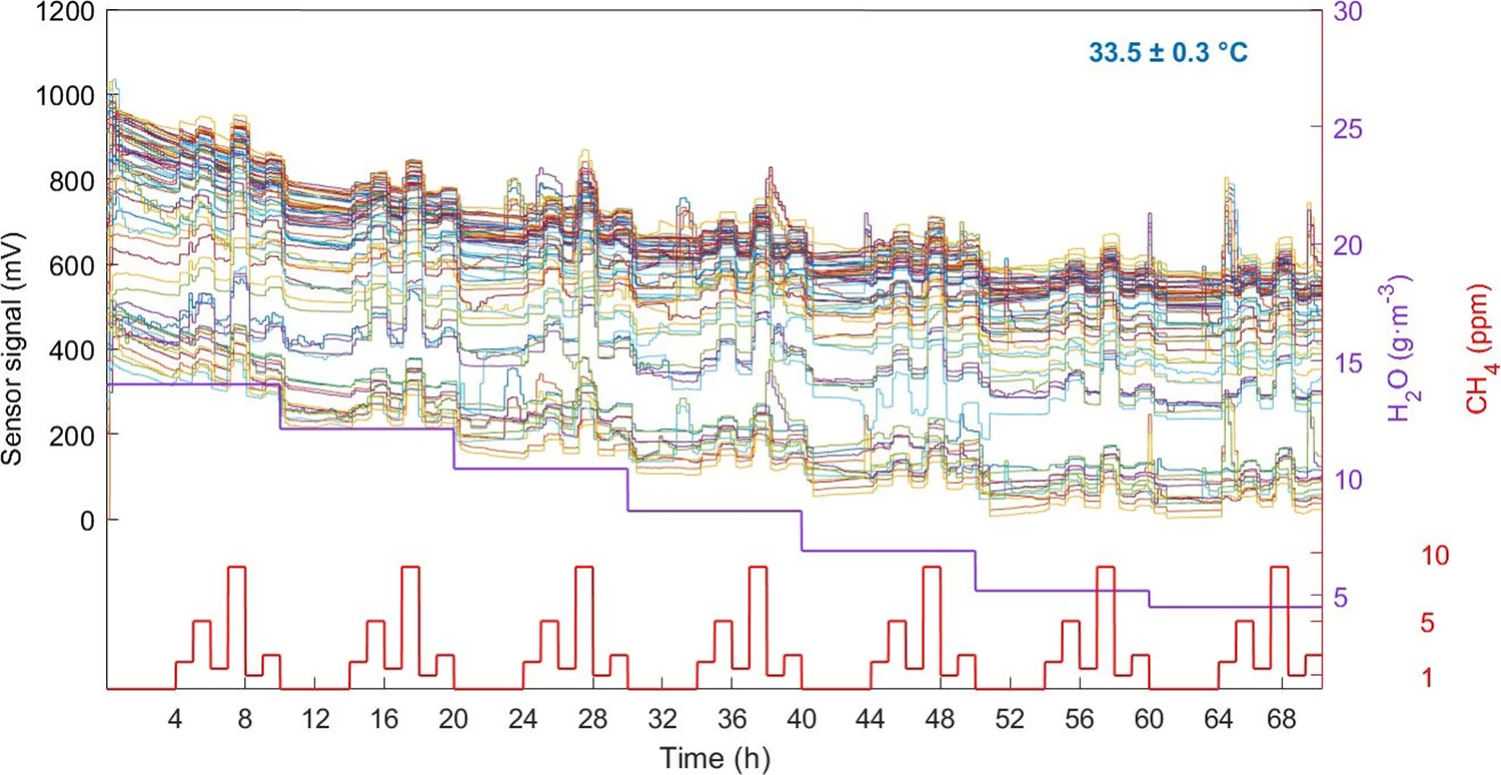
Thin lines of different colors correspond to the temporal evolution of the signal of 65 TGS2611-E00 sensors at 33.5 ± 0.3 °C when exposed to different concentrations of methane ranging from 0 to 9 ppm (thick red line) and water vapor ranging from 4.5 to 14.0 g·m^–3^ (thick purple line).

We studied the signal of 35 TGSC and 70 TGSE sensors with the GMS. Of these, three TGSC and two TGSE reported negative values, two TGSE reported a high level of noise, and one TGSE showed only response to H_2_O(g). Therefore, data from these sensors were discarded. The signals acquired for the 65 remaining TGSE and the 32 remaining TGSC, together with the *T*, H_2_O(g), and CH_4_ inside the gas test chamber, are shown in Figures 2 and S4, respectively.

**Figure 3:**
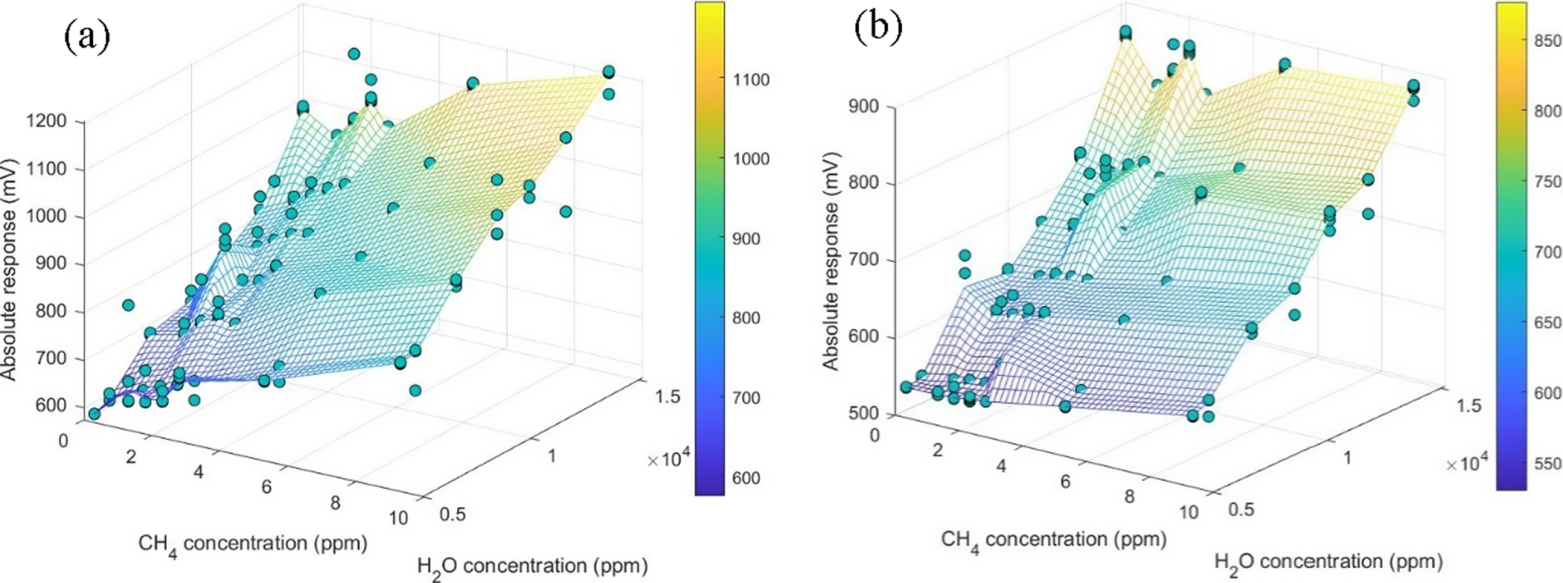
Sensor response magnitude summary for different concentrations of methane ranging from 0 to 9 ppm and water vapor ranging from 4.5 to 14 g·m^–3^ of a (a) TGS2611-C00 and a (b) TGS2611-E00 sensor.

The sensor signal increases, as expected for an n-type metal oxide like SnO_2_,^20^ when the sensors are exposed to reducing gases such as CH_4_ and H_2_O(g), while it decreases when the gas is removed from the gas test chamber. Furthermore, at the CH_4_ and H_2_O(g) concentrations tested in this work, increasing concentration gives higher voltage values, while saturation was not observed. Each individual sensor showed different behaviors, as there were different baselines and response patterns among them (Figure 2). Similarly, Figure S6a illustrates a different behavior of the sensors TGSE when compared to that of TGSC. The key differences between sensor types are illustrated in Figure 3, where the response magnitude is plotted as a function of the CH_4_ and H_2_O(g) concentrations for a TGSC and a TGSE sensor. In Figure 3 (Figure S5, 2D facets), we can observe that CH_4_ has a higher influence on the signal of the TGSC sensors than it has in the signals of the TGSE sensors by comparing the steepness of the voltage as a function of CH_4_ concentration. Instead, H_2_O(g) produces a higher inclination in the voltage of the TGSE sensors than in the case of the TGSC sensors. When comparing the effects of both gases on the overall sensor signal, we can see that CH_4_ promotes smaller changes than H_2_O(g) and that this effect is more pronounced for the TGSE sensors. This data evaluation was performed with all of the characterized sensors, obtaining the same trends. These results indicate that the gas filter included in the TGSE may influence the interaction with both studied gases, such as slowing down the reaction rate to CH_4_ as well as decreasing the H_2_O(g) removal rate.

We observed certain irregularities at values around 2 ppm of CH_4_ (see the bump close to this concentration for both types of sensors in Figure 3 and for TGSE also in Figure S5) as well as larger response for lower concentrations or unexpected peaks for few of the measurements (Figures 2 and S4) that were discarded. We attribute this behavior around 2 ppm of CH_4_ to particularities of the GMS, which were observed as well when the system was calibrated with the GC (Figure S1), while larger response than expected or unpredicted peaks are attributed to gas supply oscillations in our facilities due to occasional renovation, refilling, or parallel use of the nitrogen and oxygen gas bottles that are shared with other research groups in the department.

Besides the initial change of the sensor signal when changing the H_2_O(g) concentration inside the gas test chamber, we observed an hours-lasting drift (Figures 2 and S4), typical in SnO_2_-based gas sensors.^21,22^ The drift decreased with both time and decreasing initial H_2_O(g) concentrations with a pattern similar to an exponential decay function (see the 4 h periods between the groups of ROIs in Figures 2 and S4). A comparison between both TGS sensor signals (Figure S6a,b) shows a smaller drift for TGSC sensors than for TGSE (Figure S6a) and an initial stabilization of both TGS sensor signals within 4 h when H_2_O(g) was kept at 1.1 g·m^–3^ (Figure S6b). The stabilization time of these sensors was more than 12 h when measurements started with H_2_O(g) concentrations of 14.0 g·m^–3^ H_2_O(g). Thus, these sensors need 4–20 h after turn-on, and this time may be influenced by H_2_O(g) levels, although present data are not enough for confident separation of time versus H_2_O(g) effects on this initial stabilization period. However, results indicate that long-term operation is desirable compared to frequent power on/off of the LCSS.

To evaluate whether the observed results could be interpreted as a simple additive behavior of gas–surface interactions of the different gases, i.e., treating the sensor responses to CH_4_ and H_2_O(g) separately, without any additional interaction effects between the gases influencing the sensor response, we performed GLM. The GLM was designed to test for both main and interaction effects between factors. The *F*_0_ and *p*-values resulting from the GLM (Table S2) revealed no statistical evidence of an interaction effect between CH_4_ and H_2_O(g) on the sensor signal. GLM also supports stronger effects for H_2_O(g) than for CH_4_, more pronounced in TGSE than in TGSC (Figure S7). The possibility of interaction effects at higher H_2_O(g), or at higher CH_4_ levels as indicated elsewhere,^5^ can however not be excluded.

### 3.2 Methane Quantification under Controlled Conditions

**Figure 4:**
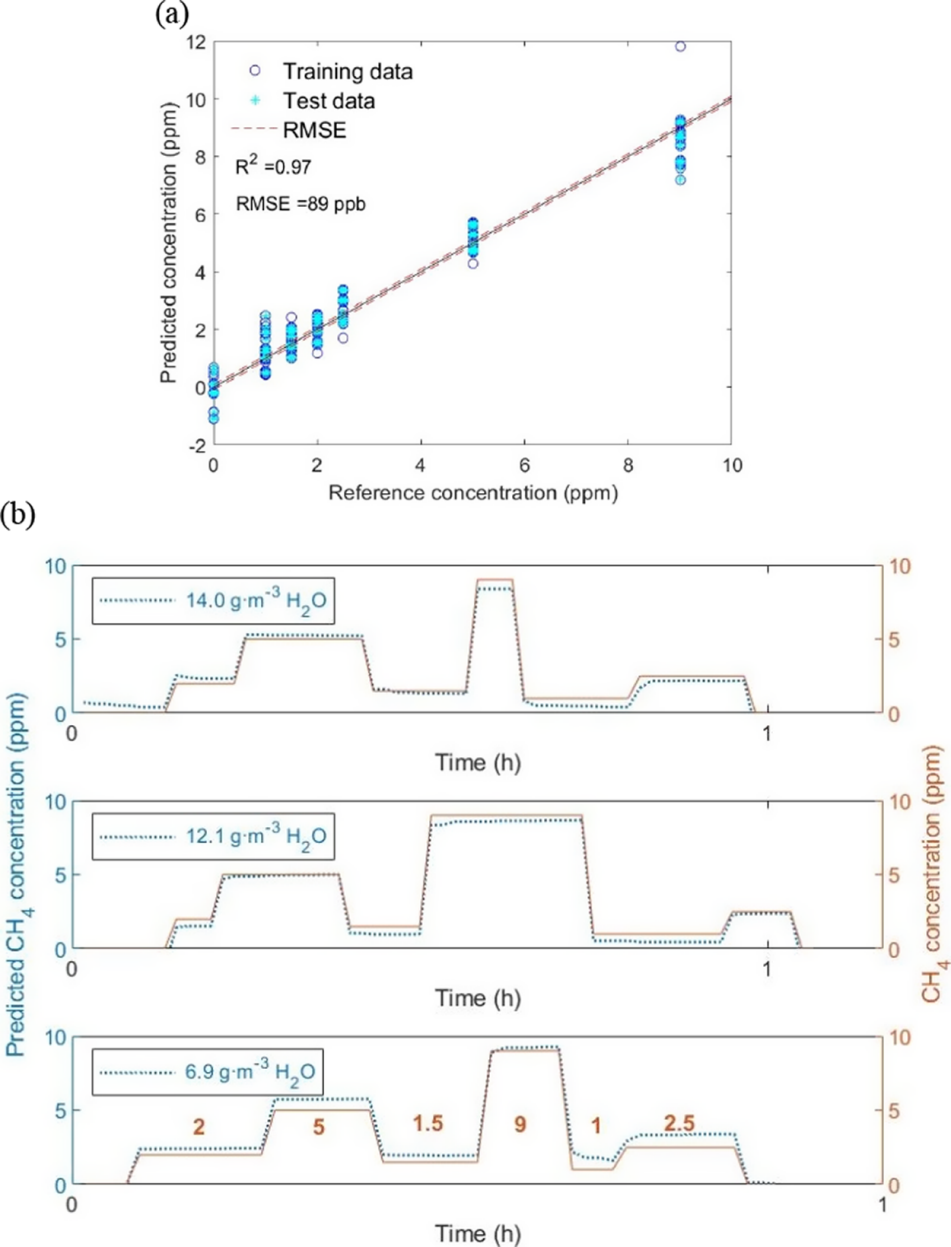
(a) Results of a partial least-squares regression for methane ranging from 0 to 9 ppm under different concentrations of water vapor ranging from 4.5 to 14.0 g·m^–3^ and (b) application of this regression to the test data (not used to fit the regression coefficients) to predict the methane concentration over time compared to the supplied concentration.

The results obtained from the cross-validated PLSR model when training the model with measurements performed in the laboratory are shown in Figure 4 as an example for one particular e-nose (the figure shows both the training points to fit the regression coefficients and the separate predictions obtained with the points reserved for testing). In this case, the model obtained a coefficient of determination, *R*^2^, of 0.97, and a RMSE of 89 ppb, while the test data correlate properly with the trained data as can be observed in Figure 4a. The same procedure was implemented to all the characterized sensors from other LCSS obtaining *R*^2^ always higher than 0.9 and RMSE always lower than 100 ppb. To test the quantification coefficients obtained for this model, we plotted the randomly selected test data (blue dotted line) predicting the CH_4_ concentration at different H_2_O(g) levels during the measurement together with the concentration introduced by means of GMS (continuous brown line) in Figure 4b. This result shows that the methodology here implemented to characterize the sensors and evaluate the data can be used to quantify CH_4_ concentrations under controlled conditions with a small RMSE (lower than 100 ppb) while overcoming the effects of H_2_O(g), which is an important issue with chemical sensors in practical applications.

The quantification coefficients obtained for this e-nose-LCSS are shown in Table S3 together with the detailed process on how to obtain the CH_4_ concentration at any H_2_O(g) concentration from the signal of the sensors (Supporting Information, Section 7). It is important to keep in mind that for each e-nose, the coefficients differ due to small differences between devices and the sensing material inducing different baselines and/or different response magnitudes to gases, as mentioned previously. However, the formula will always maintain the same shape, as follows:
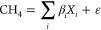
1where CH_4_ is the Z-standardized reference methane concentration (the *Y*-matrix data), β_*i*_ are the quantification coefficients corresponding to the *i* predictor obtained when training the PLSR model, *X*_*i*_ are the different Z-standardized features chosen to train the PLSR model (in this case, the ROI of the signals from one LCSS), and ε is the constant term.

### 3.3 Methane Quantification in the Field

**Figure 5:**
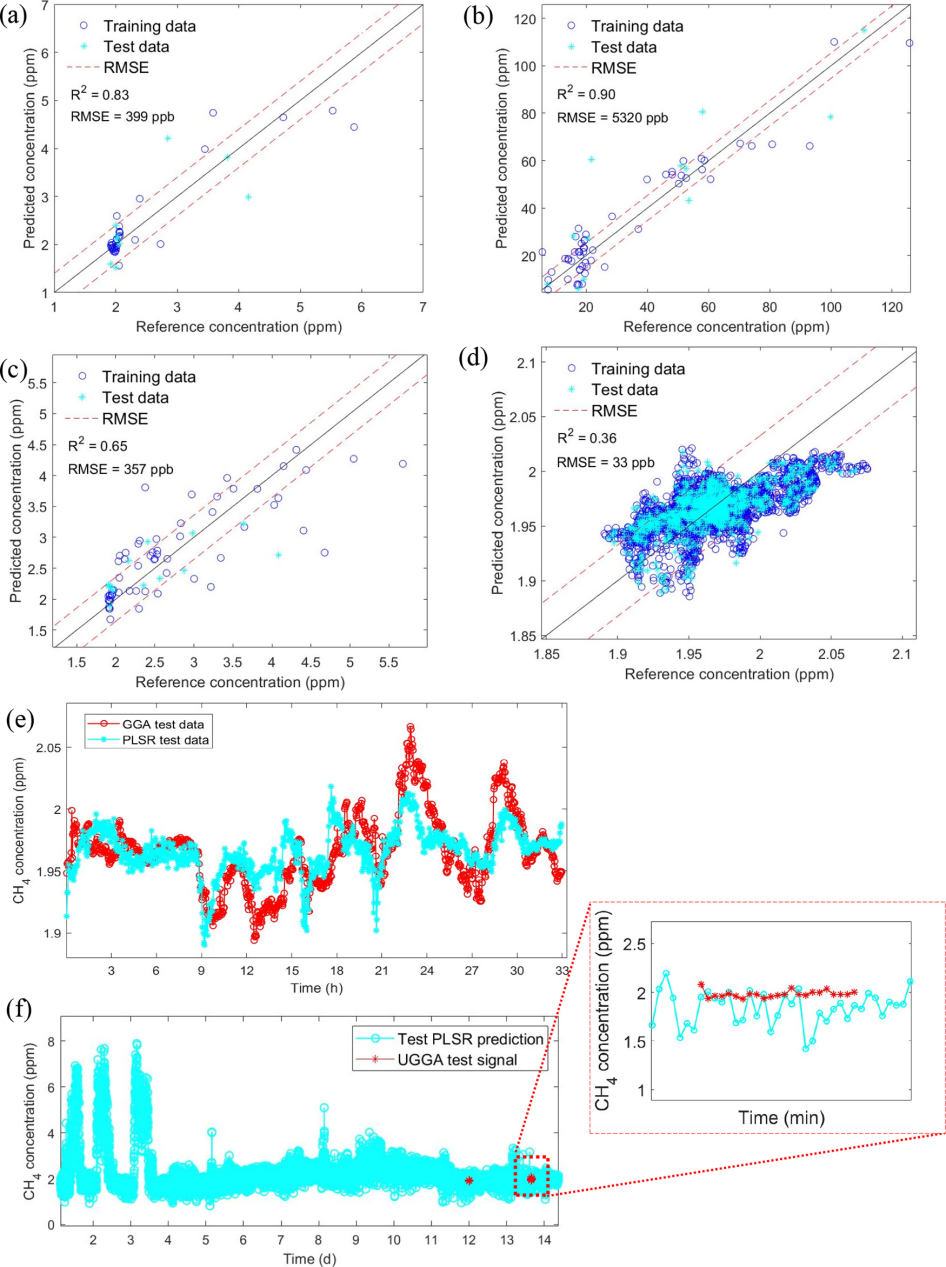
Results from partial least-squares regression models trained and tested with data acquired in the field sites corresponding to (a) wetland, (b) sludge screw room, (c) sludge piles, and (d) garden. Reference data versus (e) data used to test the quantification model from garden (d) and (f) temporal predictions of methane concentration calculated with the quantification model obtained in wetland (a). *R*^2^ in this figure is *R*_train_^2^. *R*_test_^2^ is shown in Table S4.

The results for the different sites of the PLSR model prepared for field measurements, including 12 features derived from the three TGS sensor signals in one LCSS, and H_2_O(g), *P*, and *T* from the BME680 as features in the predictor matrix and CH_4_ reported by GGAs in the response matrix are shown in Figure 5. The test data calculated with the quantification coefficients resulting from the training data fit the trends of the reference GGA data well at all of the different studied sites, albeit with different accuracies. Depending on the case studied, the *R*^2^ varies from 0.36 to 0.91 and the RMSE from 33 ppb to 5.3 ppm in proportion to the CH_4_ variability range (Table S4). For example, the greatest RMSE of 5.3 ppm corresponded to a concentration variability of more than 126 ppm, yielding a relative error of 4.5%. The variability in *R*^2^ is also partly related to the concentration range used to train the model. Wider concentration ranges typically yield higher *R*^2^, while narrower ranges yield lower *R*^2^ because of the intrinsic feature of *R*^2^ to approach zero even if models are accurate and report values close to the mean of the reference data. Therefore, RMSE is often a better accuracy indicator when comparing data sets having different concentration ranges. In addition, Figure 5, Tables S4, and S12 show that RMSE is related to the amount of reference data available. With more limited training data, the model capacity to follow CH_4_ concentration variability tends to be reduced (greater RMSE). This is clear from comparing the garden data, rich in reference measurements and consequently superior model performance (lower RMSE) in spite of being a demanding study case with small ambient atmospheric concentration fluctuations (Figure 5e), versus the wetland data, model with more limited reference data that enables identifying the baseline concentration level but not following the atmospheric fluctuations (Figure 5f).

For the field measurements, examples of quantification coefficients and additional information obtained for each site are shown as examples in Table S4. In this case, the formula to calculate the CH_4_ concentration is (1), mentioned in Section 3.2. However, we obtain 12 β_*i*_ coefficients because we use 12 *X* variables to train the model. Again, different e-nose-LCSSs give different coefficients due to differences among constituting devices.

**Figure 6:**
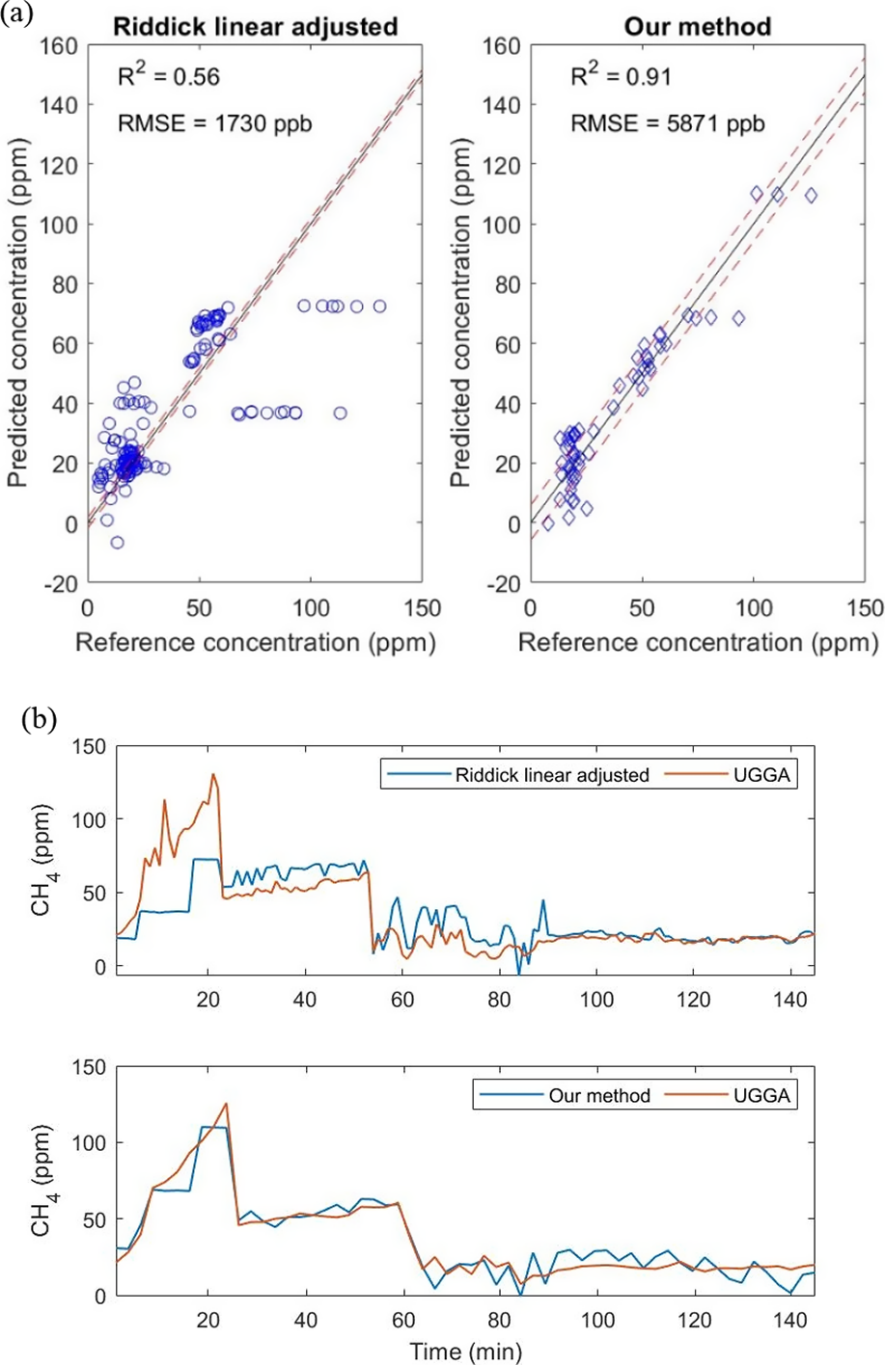
(a) Comparison between the best results obtained among the previous methods studied when quantifying methane and our method and (b) temporal evolution of (a).

Previous attempts to calibrate TGS sensors for outdoor open atmospheric measurements were compared to our approach by using all data from the wetland, sludge screw room, sludge piles, and garden sites data sets (Table S5 and Figures S8–S11). In this comparison, we used selected previously published calibration models on our data in two ways. First, we used the original published calibration equation coefficients. Then we just used the published calibration model equations but fitted the coefficients to our data. While several of the previous approaches reported similar performance for their respective original data as we present here for our e-nose approach, the e-nose approach seemed more promising when applied to the data sets presented here. Figure 6 provides an example of a comparison between the two best-performing models in this comparison. It is important to consider that sensor evaluations are somewhat biased and perform better on the original data used for their development when the models are tested with the same training data. Hence, model intercomparisons are important and should be given more attention in future work. Further, it is clear that monitoring atmospheric concentration fluctuations at high temporal resolution is challenging with this type of sensor. Notable, both this work and the study of Eugster et al.^4^ reached similar performance with different models for their respective atmospheric measurements (Arctic air versus garden data), and in both cases, the TGS sensors underestimated the highest peaks detected by the reference instruments (e.g., Figure 5e and Figures 5–8 in Eugster et al.^4^). This indicates the possibility of a systematic bias between reference data and TGS sensor measurements that deserve further attention. Overall, while past models can be favorable in requiring less information or less data processing, the e-nose approach presented seems highly useful in offering a general model that can integrate large and variable amounts of information and easily be extended to improve or optimize sensor calibration models, yet in reasonably transparent ways. This successful demonstration of the approach in multiple measurements contexts with variable CH_4_ ranges and other conditions and with variable amounts of data indicates that the e-nose approach offers a promising solution to reach high accuracy and sensitivity combined with high versatility and cost-effectiveness.

The presented e-nose methodology enabled the monitoring of methane at ambient atmospheric levels (around 2 ppm: given sufficient reference data) and higher (up to a few tens of ppm), with three TGS-type sensors and one BME680 (total system cost of about €500). Root-mean-square errors down to 33 ppb, *R*^2^ up to 0.91, yielded useful temporal predictions when compared to reference equipment (with costs being 2 orders of magnitude higher) in laboratory and field applications. The accuracies obtained and the variability of cases studied highlight the predictive versatility of the e-nose approach that deserves further attention and has a high potential for some types of CH_4_ monitoring. Although TGS sensors show cross-sensitivity to humidity and their response is likely affected by surrounding temperature, atmospheric pressure, and the presence of other reduced trace gases, the developed systematic procedure based on the e-nose seems to offer ways to compensate, at least partially, such interfering parameters. While laboratory calibrations are useful to characterize sensor responses and cross sensitivities, such calibrations cannot yet provide the calibration covering the full range of interfering parameters in the field. Therefore, periodic field calibration with reference equipment is necessary to maximize in situ accuracy and reliability. The e-nose approach can accommodate both laboratory and in situ calibrations using the same fundamental model, enabling reliable LCSS networks supported by recurring reference measurements. The amount of reference measurements and the local gas concentration ranges and variability are important for measurement accuracy and need consideration in measurement design. Given appropriate model training of the quantification models with adequate amounts of data matching desired accuracy, on-board processing of the sensor signals can be designed for direct reporting of methane concentrations. Thus, the work presented here is encouraging for the future and for a more widespread use of networks of LCCCs for the monitoring of greenhouse gases.

## Supporting Information Available

Reference equipment calibration, humidity and temperature study inside the gas test chamber, sticker labels study, regions of interest and feature calculation details, additional sensor characterization, analysis of variance details, partial least-squares regression example coefficients and procedure, and former methods comparison, and accuracy of the study (PDF)
Data file from the measurements in Section 3.2 (XLSX)
Data file from the wetland measurements in Section 3.3 (XLSX)
Data file from the sludge screw room measurements in Section 3.3 (XLSX)
Data file from the sludge piles measurements in Section 3.3 (XLSX)
Data file from the garden measurements in Section 3.3 (XLSX)

## References

[ref1] Lan X.; Thoning K. W.; Dlugokencky E. J. Trends in globally-averaged CH4, N2O, and SF6 determined from NOAA Global Monitoring Laboratory measurements. 2022, Version 2023–03. 10.15138/P8XG-AA10.

[ref2] Myhre G.; Shindell D. Anthropogenic and Natural Radiative Forcing. In Climate Change 2013: The Physical Science Basis. Contribution of Working Group I to the Fifth Assessment Report of the Intergovernmental Panel on Climate Change; Stocker T., Qin D., Plattner G.-K., Tignor M., Allen S., Boschung J., Nauels A., Xia Y., Bex V., Midgley P., Eds.; Cambridge University Press, 2013; pp 659–740.

[ref3] Saunois M.; Stavert A. R.; Poulter B.; Bousquet P.; Canadell J. G.; Jackson R. B.; Raymond P. A.; Dlugokencky E. J.; Houweling S.; Patra P. K.; Ciais P.; Arora V. K.; Bastviken D.; Bergamaschi P.; Blake D. R.; Brailsford G.; Bruhwiler L.; Carlson K. M.; Carrol M.; Castaldi S.; Chandra N.; Crevoisier C.; Crill P. M.; Covey K.; Curry C. L.; Etiope G.; Frankenberg C.; Gedney N.; Hegglin M. I.; Höglund-Isaksson L.; Hugelius G.; Ishizawa M.; Ito A.; Janssens-Maenhout G.; Jensen K. M.; Joos F.; Kleinen T.; Krummel P. B.; Langenfelds R. L.; Laruelle G. G.; Liu L.; Machida T.; Maksyutov S.; McDonald K. C.; McNorton J.; Miller P. A.; Melton J. R.; Morino I.; Müller J.; Murguia-Flores F.; Naik V.; Niwa Y.; Noce S.; O’Doherty S.; Parker R. J.; Peng C.; Peng S.; Peters G. P.; Prigent C.; Prinn R.; Ramonet M.; Regnier P.; Riley W. J.; Rosentreter J. A.; Segers A.; Simpson I. J.; Shi H.; Smith S. J.; Steele L. P.; Thornton B. F.; Tian H.; Tohjima Y.; Tubiello F. N.; Tsuruta A.; Viovy N.; Voulgarakis A.; Weber T. S.; van Weele M.; van der Werf G. R.; Weiss R. F.; Worthy D.; Wunch D.; Yin Y.; Yoshida Y.; Zhang W.; Zhang Z.; Zhao Y.; Zheng B.; Zhu Q.; Zhu Q.; Zhuang Q. The Global Methane Budget 2000–2017. Earth Syst. Sci. Data 2020, 12, 1561–1623. 10.5194/essd-12-1561-2020.

[ref4] Eugster W.; Laundre J.; Eugster J.; Kling G. W. Long-term reliability of the Figaro TGS 2600 solid-state methane sensor under low-Arctic conditions at Toolik Lake, Alaska. Atmos. Meas. Technol. 2020, 13, 2681–2695. 10.5194/amt-13-2681-2020.

[ref5] Bastviken D.; Nygren J.; Schenk J.; Massana R. P.; Duc N. T. Technical note: Facilitating the use of low-cost methane (CH_4_) sensors in flux chambers – calibration, data processing, and an open-source make-it-yourself logger. Biogeosciences 2020, 17, 3659–3667. 10.5194/bg-17-3659-2020.

[ref6] Methane emission measurement and monitoring methods. In: Improving characterization of anthropogenic methane emissions in the United States; National Academy of Sciences, Ed.; National Academy Press, 2018; pp 77–138. 30110140

[ref7] Bastviken D.; Wilk J.; Duc N. T.; Gålfalk M.; Karlson M.; Neset T.-S.; Opach T.; Enrich-Prast A.; Sundgren I. Critical method needs in measuring greenhouse gas fluxes. Environ. Res. Lett. 2022, 17, 104009 10.1088/1748-9326/ac8fa9.

[ref8] Shah A.; Laurent O.; Lienhardt L.; Broquet G.; Rivera Martinez R.; Allegrini E.; Ciais P. Characterising Methane Gas and Environmental Response of the Figaro Taguchi Gas Sensor (TGS) 2611-E00. Atmos. Meas. Technol. Discuss. 2023, 16, 3391–3419. 10.5194/amt-2022-308.

[ref9] Rivera Martinez R.; Santaren D.; Laurent O.; Cropley F.; Mallet C.; Ramonet M.; Caldow C.; Rivier L.; Broquet G.; Bouchet C.; Juery C.; Ciais P. The Potential of Low-Cost Tin-Oxide Sensors Combined with Machine Learning for Estimating Atmospheric CH_4_ Variations around Background Concentration. Atmosphere 2021, 12, 107 10.3390/atmos12010107.

[ref10] Park S. Y.; Kim Y.; Kim T.; Eom T. H.; Kim S. Y.; Jang H. W. Chemoresistive materials for electronic nose: Progress, perspectives, and challenges. InfoMat 2019, 1 (3), 289 10.1002/inf2.12029.

[ref11] Product information: TGS 2611-C00 - for the detection of Methane; rev. 03/21; https://www.figarosensor.com/product/entry/tgs2611-c00.html (accessed March 2023).

[ref12] Product information: TGS 2611-E00 - for the detection of Methane; rev 08/22; Technical note: Technical Information for Methane Gas Sensors; rev. 08/22; Application note: Application Notes for Methane Gas Detectors using TGS2611-E00; rev 04/22; https://www.figarosensor.com/product/entry/tgs2611-e00.html#ti (accessed March 2023).

[ref13] BME680 Datasheet: Low power gas, pressure, temperature & humidity sensors; BST-BME680-DS001–00; 1 277 340 511; rev. 1.0; July, 2017. https://www.bosch-sensortec.com/products/environmental-sensors/gas-sensors/bme680/#documents (accessed March 2023).

[ref14] SHTC1 Datasheet; rev. 5; December, 2022. https://sensirion.com/products/catalog/SHTC1/ (accessed March 2023).

[ref15] LGR-ICOS GLA131 Series, Microportable Greenhouse Gas Analyzers Datasheets; DS/LGR-ICOS/MGGA-EN; rev. D; October, 2019. http://www.lgrinc.com/analyzers/overview.php?prodid=43&type=gas (accessed March 2023).

[ref16] Montgomery D. C. Design and analysis of experiments, 5th edition; John Wiley Sons Inc., 2001; pp 1–119.

[ref17] Wold S.; Sjostrom M.; Eriksson L. PLS-Regression: A Basic Tool of Chemometrics. Chemom. Intell. Lab. Syst. 2001, 58, 109–130. 10.1016/S0169-7439(01)00155-1.

[ref18] Hastie T.; Tibshirani R.; Friedman J. Overview of supervised learning & Model Assessment and Selection. In The Elements of Statistical Learning, 2nd edition; Springer, 2008; pp 1–39.

[ref19] Kvålseth T. O. Cautionary Note about R^2^. Am. Stat. 1985, 39 (4), 279–285. 10.1080/00031305.1985.10479448.

[ref20] Domènech-Gil G.; Samà J.; Fàbrega C.; Gràcia I.; Cané C.; Barth S.; Romano-Rodríguez A. Highly sensitive SnO_2_ nanowire network gas sensors. Sens. Actuators B Chem. 2023, 383, 133545 10.1016/j.snb.2023.133545.

[ref21] Clifford P. K.; Tuma D. T. Characteristics of semiconductor gas sensors I. Steady state gas response. Sens. Actuators 1982, 3, 233–254. 10.1016/0250-6874(82)80026-7.

[ref22] Watanabe K.; Ohgaki T.; Saito N.; Hishita S. Interaction of Water Vapor with SnO_2_. In Book of abstracts, The 14th International Meeting on Chemical Sensors (IMCS 2012), Nurembergx, GE.

