# Supplementary material for: Electronic Nose for Improved Environmental Methane Monitoring
Source: Environ Sci Technol. 2023 Dec 21;58(1):352–61. doi: 10.1021/acs.est.3c06945 (PMC10785752; doi:10.1021/acs.est.3c06945)
Supplement: Supplementary file 1 — es3c06945_si_001.pdf [file es3c06945_si_001.pdf]

An electronic nose for improved environmental methane monitoring – Supplementary Information

Guillem Domènech-Gil[1], Nguyen Thanh Duc[1], J. Jacob Wikner[2†], Jens Eriksson[3], Sören Nilsson Påledal[4], Donatella Puglisi[3], David Bastviken[1*]*

[1] Department of Thematic Studies and Environmental Change (TEMA M), Linköping University, Linköping 58183, Sweden

[2] Department of Electrical Engineering (ISY), Linköping University, Linköping 58183, Sweden

[3] Department of Physics, Chemistry, and Biology (IFM), Linköping University, Linköping 58183, Sweden

[†] GE Healthcare, Teknikringen 8, Linköping 58330, Sweden (present address)

[*] Corresponding authors' email: guillem.domenech@liu.se, david.bastviken@liu.se.

33 pages (including cover page)

5 Tables (S1, S2, S3, S4, S5)

11 Figures (S1, S2, S3, S4, S5, S6, S7, S8, S9, S10, S11)

1. REFERENCE EQUIPMENT

The results of the gas mixing system (GMS) and the ultra-portable greenhouse gas analyzer (UGGA) calibration using the gas chromatograph (GC) are shown in Figure S1. Mean uncertainties relative to the GC are included in the graph.

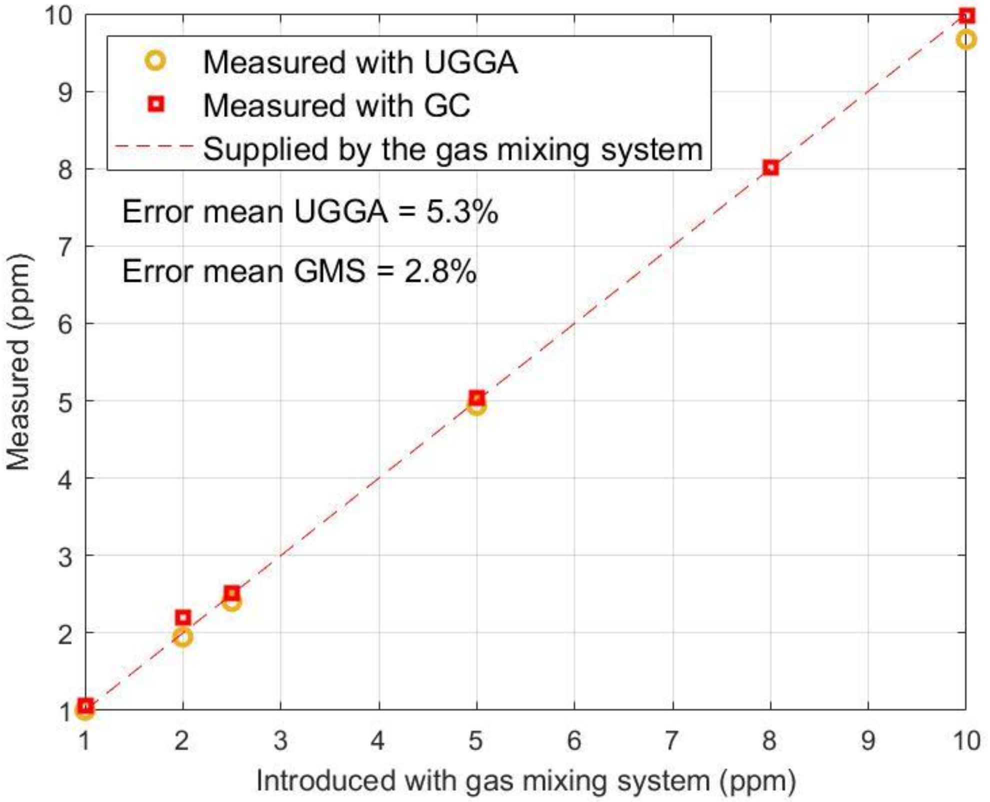

Figure S1. Methane concentration supplied by our gas mixing system and the corresponding concentrations measured with an ultraportable greenhouse gas analyser and a gas chromatograph.

2. HUMIDITY AND TEMPERATURE STUDY INSIDE THE GAS TEST CHAMBER

The water vapor ($H_2O(g)$) concentration and temperature (T) along the gas test chamber reported by the SHTC1 sensor before starting the calibration measurements at three different points (introduced gas, in the chamber near the inlet and at the central part of the chamber) are summarized in Table S1. While introducing 15.3 g·m^{-3} (76.0% RH at 22.6 °C) by means of the GMS, the SHTC1 reported a mean value of 15.5 g·m^{-3} (35.6% RH at 37.0 °C) inside

the chamber. Thus, we found similar $H_2O(g)$ concentration before and along the chamber; about 15.4 g·m^{-3}, with errors below 4%.

Table S1. Gas chamber study

Parameter	Introduced	Inlet	Central
T (°C)	22.6 ± 0.1	36.0 ± 0.5	38.0 ± 0.5
RH (%)	76.0 ± 0.2	38.0 ± 0.2	33.2 ± 0.2
$H_2O(g)$ (g·m^{-3})	15.3	15.8	15.3
$H_2O(g)$ error (%)		3.3	0.1

Notes: Relative humidity and temperature values measured with a SHTC1 before and along the gas test chamber when the gas mixing system was supplying 76% of relative humidity, and the corresponding $H_2O(g)$ concentrations calculated at 1018 hPa.

3. STICKER LABELS STUDY

The results of the two measurements, with and without sticker labels, were compared and no relevant difference was observed after the sensor stabilization period, see Figure S2. We conclude that the sticker labels used did not influence the measurements.

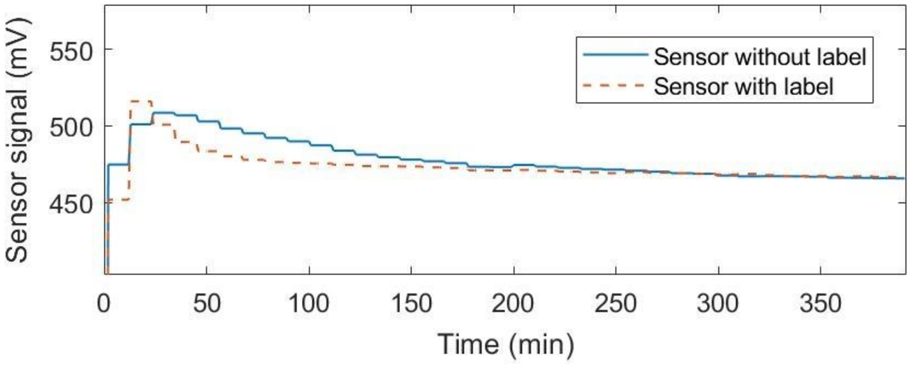

Figure S2. Temporal signal evolution of a TGS2611-E00 sensor without and with sticker label showing no relevant differences after stabilization.

4. REGIONS OF INTEREST AND FEATURE CALCULATION DETAILS

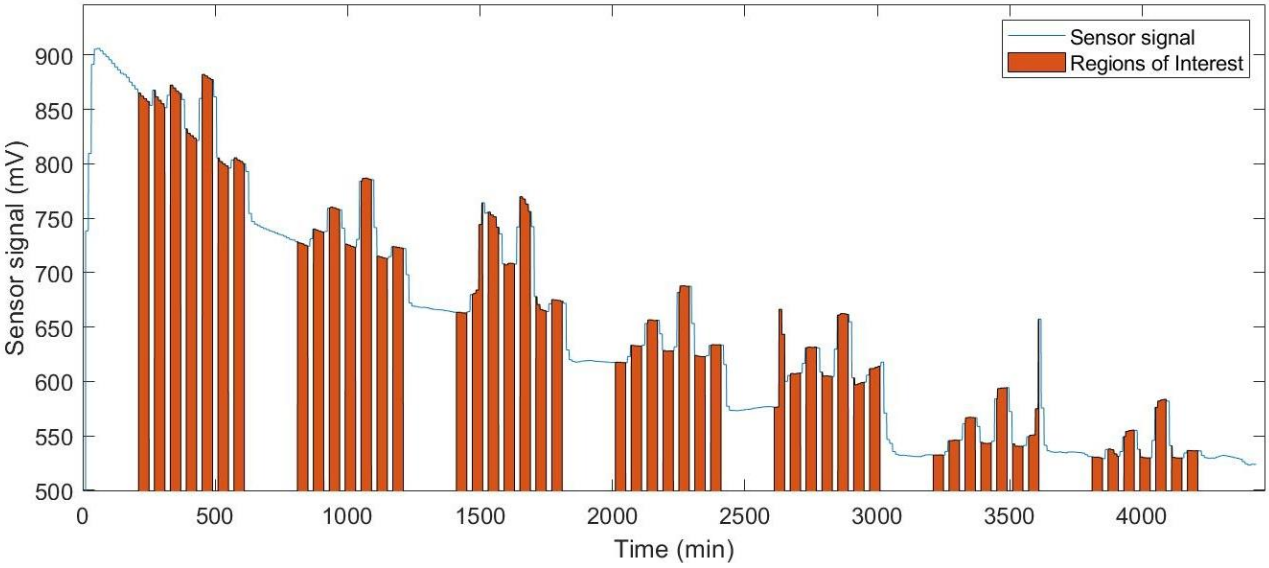

Figure S3. Example of regions of interest from a measurement performed in the laboratory with the gas mixing system showing the signal of a TGS sensor that was exposed to seven CH_4 concentrations (0 to 9 ppm) repeatedly over seven H_2O(g) concentrations (4.5 to 14 g·m^{-3}). The regions of interest, containing the data selected to elaborate CH_4 quantification models, are marked and denote time windows during each combination of CH_4 and H_2O(g) concentrations.

The three types of two-minute intervals features used for sensor signal evaluation (mean value, slope, and fast Fourier transform, chosen because they are not mathematically dependent from each other and, therefore, reporting new and different types of information from the sensor signal, were calculated in the following way:

1. *Mean value*: summing up the value of the sensor signal at minute i and minute $i+1$, and dividing by two.

2. *Linear slope*: between the sensor signal *(y)* at minute i and minute *$i+1$, and time (t),* i.e., the steepness of the sensor signal $m = \Delta y/\Delta x$.

3. *Fast Fourier transform (FFT)*: summing up the value of the sensor signal at minute *i* and minute *i+1*, dividing by two, and then converting the sensor signal from time domain to frequency domain using the FFT algorithm[1] in Matlab software. Later, calculating its absolute value, and calculating its natural logarithm.

The relevance of each feature depends on each case studied. This was tested by calculating the variable importance in projection (VIP) scores. The VIP scores measure the contribution of each variable to the model and can be used to rank the variables by their importance. Usually, the variables with VIP scores higher than 1 are the most important. We obtained values higher than 1 differently in each of the cases we studied, informing of more relevance for some of the features or for some others depending on the particularities of each situation. The area under the sensor signal and the difference between consecutive values were tested as well and reported similar regression coefficients as the mean value and slope, respectively. For this reason and to avoid overfitting, these two extra features were discarded. For the three used features, we cannot extract a conclusion of which are the most relevant features, but that all of them were important to train models that could adapt to different scenarios.

5. ADDITIONAL SENSOR CHARACTERIZATION

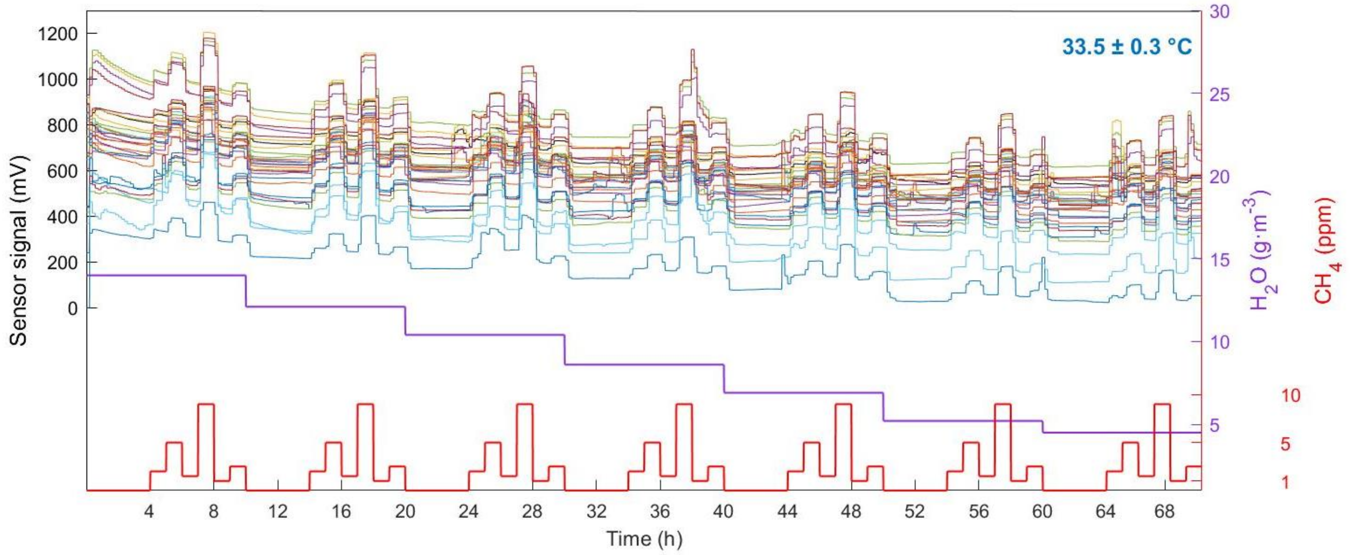

Figure S4. Temporal evolution of the signal of 32 TGS2611-C00 sensors exposed to different concentrations of methane, ranging from 1 to 9 ppm, and water vapor, ranging from 4.5 to 14 g·m^{-3}, at 33.5 ± 0.3 °C.

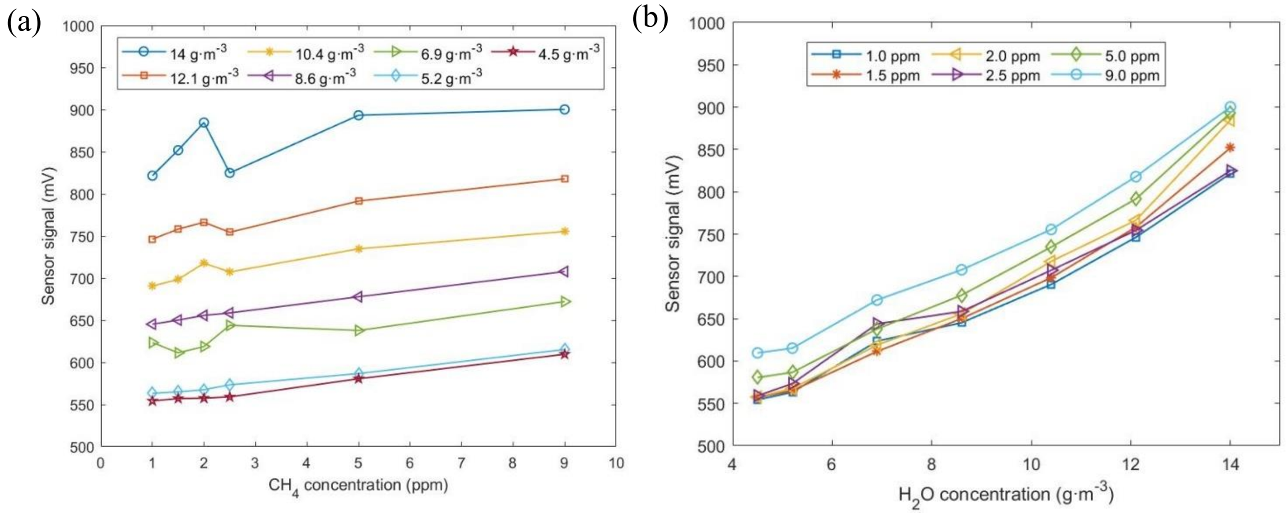

Figure S5. Summary of the sensor signal for different concentrations of methane, ranging from 0 to 9 ppm, and water vapor, ranging from 4.5 to 14 g·m^{-3} of a TGS2611-E00 sensor as function of (a) methane; and (b) water vapor.

The results shown in Figure S5 present a linear trend for increasing CH_4 and $H_2O(g)$ concentrations except for values around 2 ppm CH_4. We attribute this behavior to particularities of the GMS at this concentration region, which were observed as well when the system was calibrated with the GC (Figure S1).

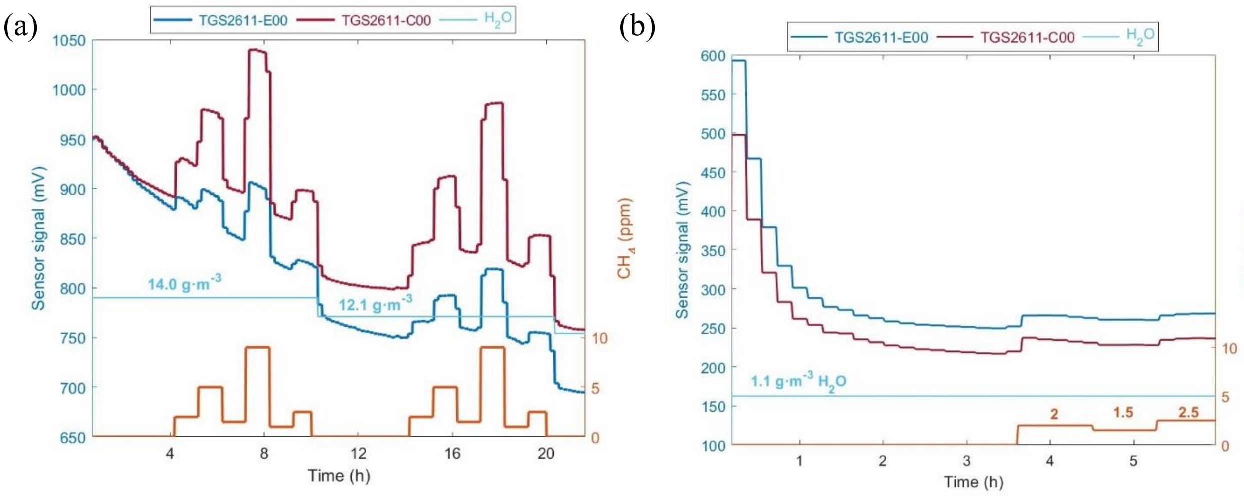

Figure S6. (a) comparison between the TGS2611-E00 and TGS2611-C00 temporal signal evolutions at 14.0, 12.1, and 10.4 g·m^{-3} of water vapor when exposed to the same concentrations of methane, and (b) TGS2611-E00 and TGS2611-C00 temporal signal evolutions with lowest water vapor concentration affordable with our gas mixing system, 1.1 g·m^{-3}, showing faster stabilization time when compared to gas mixtures with higher water vapor concentrations.

We observe a stabilization time longer than 12 h when measurements start with H_2O(g) concentrations of 14.0 g·m^{-3} H_2O(g) (Figure S6a). The baseline drift during stabilization is lower for TGSC than for TGSE sensors (Figure S6a). Calibrations starting with 1.1 g·m^{-3} (3.0% RH at 33.5 °C) show a stabilization of the signals of both type of sensors below 4 h (Figure S6b). Therefore, the sensing material itself needs below 4 h to report a stable signal after initialization of the sensor but this time increases with increasing H_2O(g) concentration.

6. GENERAL LINEAR MODEL

Table S2. GLM

**TGS2611-C00**	SS	df	MS	F_0	p-value
H_2O(g)	$9.9 \cdot 10^6$	6	$1.6 \cdot 10^6$	24.78	0
CH_4	$1.7 \cdot 10^6$	6	$2.9 \cdot 10^5$	4.36	0.0002
Interaction	$9.2 \cdot 10^5$	36	$2.6 \cdot 10^4$	0.38	0.9997
Error	$1.1 \cdot 10^8$	1617	$6.7 \cdot 10^4$		
Total	$1.2 \cdot 10^8$	1665			
**TGS2611-E00**					
H_2O(g)	$2.4 \cdot 10^7$	6	$4.0 \cdot 10^6$	116.31	0
CH_4	$9.8 \cdot 10^5$	6	$1.6 \cdot 10^5$	4.77	0.0001
Interaction	$4.9 \cdot 10^5$	36	$1.4 \cdot 10^4$	0.4	0.9995
Error	$1.1 \cdot 10^8$	3136	$3.5 \cdot 10^4$		
Total	$1.3 \cdot 10^8$	3184			

Notes: Results of the two-way analysis of variance of the absolute response of the two types of TGS sensors at seven different water vapor concentrations and seven different methane concentrations. *SS* is the sum of squares, *df* the degrees of freedom, *MS* the mean square, F_0 the values of a statistical test with an F-distribution, and *p-value* the probability that the test statistic will take on a value that is at the least as extreme as the observed value of the statistics.

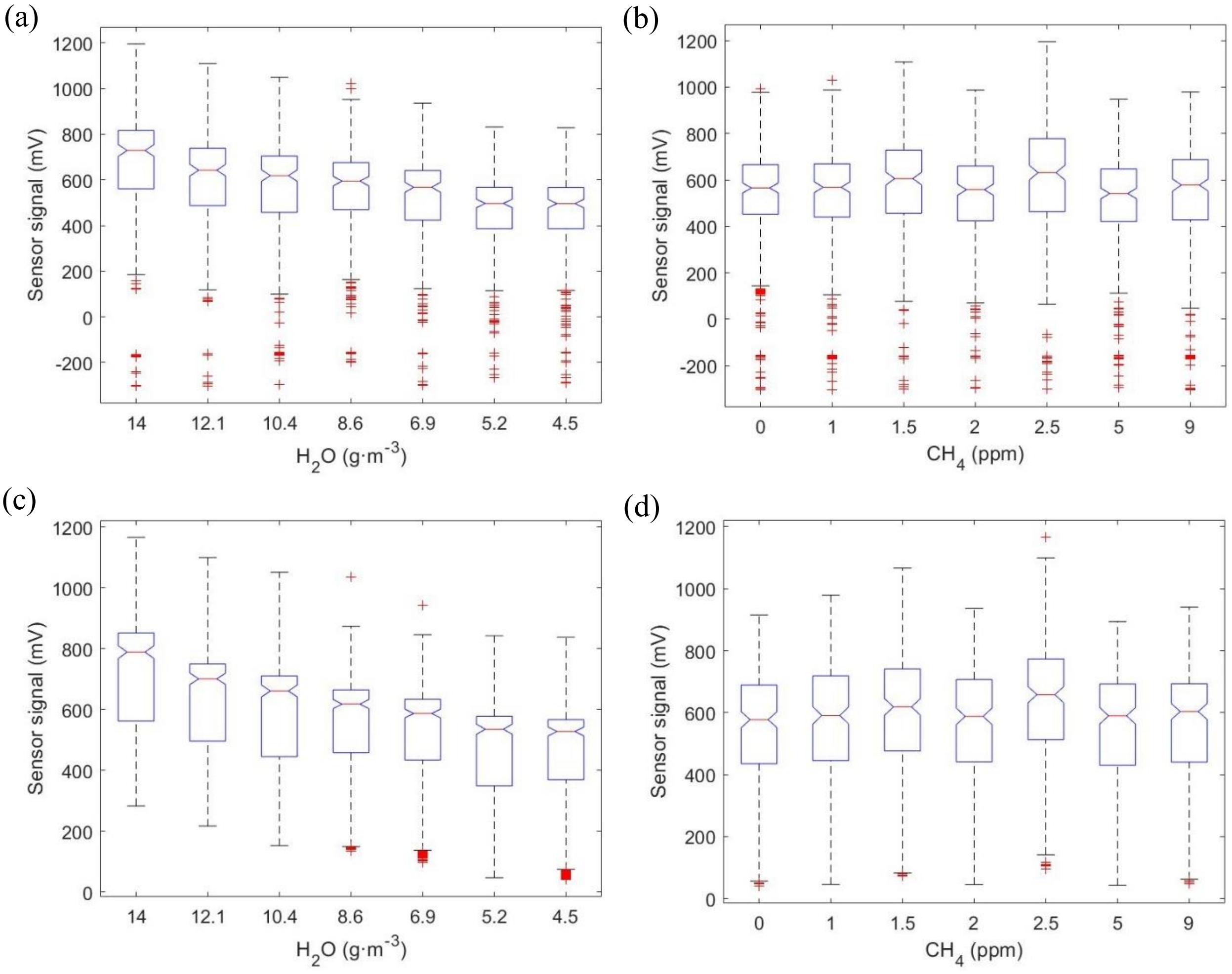

Figure S7. Boxplot results of analysis of variance showing the differences in the sensor signal as a function of water vapor and methane, respectively, for a: (a) and (b) TGS2611-C00 sensor; and (c)and (d) TGS2611-E00 sensor.

7. PARTIAL LEAST SQUARES REGRESSION EXAMPLE COEFFICIENTS AND PROCEDURE

The PLSR quantification coefficients obtained for one specific e-nose with the laboratory measurements are summarized in the following Table S3:

Table S3. Example of PLSR laboratory coefficients

β_{H2Og}	$\beta_{sensor1}$	$\beta_{sensor2}$	$\beta_{sensor3}$	ε
$\mathbf{-0.0143}$	0.6912	2.2889	-2.5763	$2.3475 \cdot 10^{-14}$

Notes: Quantification coefficients and constant term for an example model trained and tested in laboratory under controlled conditions (data shown in Figure 4). Sensor1 and Sensor3 refer to TGS2611-E00 sensors, Sensor2 to TGS2611-C00 sensor, and H_2O(g) to the humidity supplied by the gas mixing system. ***Importantly, note that these coefficients are only valid for specific LCSS and cannot be used for other sensors or LCSS.***

As an example on how to use the PLSR coefficients, we will use the standardized values of H_2O(g) of 14 g·m^{-3} and the three standardized sensor signal voltage values in the ROI for 2.5 ppm CH_4 (we choose the point 60 from the 280 available from this region) i.e., the predictor variables that will give us the CH_4 concentration (symbols explained in main text):

$$CH_4 = \beta_{H2O(g)} \cdot X_{H2O(g)} + \beta_{Sensor1} \cdot X_{Sensor1} + \beta_{Sensor2} \cdot X_{Sensor2} + \beta_{Sensor3} \cdot X_{Sensor3} + \varepsilon \quad (2)$$

$$[CH_4] = (CH_4 \cdot \sigma_{Res.train.}) + \overline{X_{Res.train.}} \quad (3)$$

$$\left.\begin{array}{l} X_{H2O(g)} = 1.5714 \\ X_{Sensor1} = 1.8749 \\ X_{Sensor2} = 1.3618 \\ X_{Sensor3} = 1.7938 \end{array}\right\} CH_4 = -0.2309$$

To convert the result into ppm, we need to multiply the result by the standard deviation of the response training data set and sum it to the mean value of the response training data set (back standardize), that for our laboratory measurements were:

$$\text{Mean value response training data set} = 3.0297\ \text{ppm}$$

$$\text{Standard deviation response training data set} = 2.8690\ \text{ppm}$$

$$[CH_4] = 2.367\ \text{ppm}$$

The PLSR quantification coefficients obtained for the laboratory measurements are summarized in the following Table S4:

Table S4. Examples of PLSR field coefficients.

**Coefficients**	**Site 1** ***Garden***	**Site 2** ***Sludge piles***	**Site 3** ***Sludge screw room***	**Site 4** ***Wetland***
$\beta_{mv\ sensor1}$	9.223	-8.212	4.510	-2.284
$\beta_{slope\ sensor1}$	0.005	-0.051	-1.087	-0.205
$\beta_{fft\ sensor1}$	-8.860	8.386	-3.553	1.852
$\beta_{H2O(g)}$	0.387	-0.154	-0.135	-0.986
$\beta_{mv\ sensor2}$	-3.713	3.064	-17.578	8.139
$\beta_{slope\ sensor2}$	$-4.949 \cdot 10^{-4}$	0.132	-0.189	0.071
$\beta_{fft\ sensor2}$	4.716	-1.850	18.844	-7.512
β_{T}	-0.375	-0.173	-1.022	-2.131
$\beta_{mv\ sensor3}$	7.8105	5.030	14.194	-0.358
$\beta_{slope\ sensor3}$	-0.032	0.050	1.212	0.171
$\beta_{fft\ sensor3}$	-9.048	-5.769	-15.987	0.463
β_{P}	0.427	-0.044	-0.135	-0.836

ε	$8.986 \cdot 10^{-14}$	$-1.954 \cdot 10^{-14}$	$-7.116 \cdot 10^{-14}$	$2.946 \cdot 10^{-13}$
$RMSE_{train}$* (ppm)	0.033	0.353	5.320	0.399
$RMSE_{test}$* (ppm)	0.001	0.131	2.895	0.042
CH_4 range (ppm)	0.186	5.202	120.6099	5.781
$RMSE_{train}$/ CH_4 range (%)	17.6	6.5	4.4	6.9
$RMSE_{test}$/ CH_4 range (%)	0.5	2.5	2.4	0.7
R_{train}^2*	0.36	0.64	0.90	0.83
R_{test}^2*	0.37	0.61	0.80	0.75
Reference data points	9898	153	145	92
Measurement duration (d)	17	140	99	30
Time measuring (%)	100	80.5	95.7	47.5

Notes: Quantification coefficients and constant terms for the partial least squares regression models trained herein and tested in the different field sites studied in our work, and the corresponding root mean squares error, methane range, percentage ratio between root mean squares error and methane range, and number of reference points available from the reference Greenhouse Gas analyzer for each field site. Sensor1 and Sensor3 are TGS2611-E00 and Sensor2 is a TGS2611-C00, while H_2O(g), T, and P are the relative humidity, temperature, and atmospheric pressure monitored by the BME680. See main text for details. ***Importantly, note that these coefficients are only valid for specific LCSS and cannot be used for other sensors or LCSS.***

*Note that $RMSE_{train}$ and R_{train}^2 correspond to the well-known measures of error calculated with the train data, while $RMSE_{test}$ and R_{test}^2 are the same measures of error but calculated with the test data. .

8. COMPARISON BETWEEN PUBLISHED APPROACHES

The response towards different gases of semiconductor materials and, in particular SnO_2, have been reported previously[2]. TGS-type sensors have existed for several decades and their response towards gases have been studied under controlled conditions in laboratory[3]. For example, the following Clifford equation have been suggested when maintaining constant current values and exposing the TGS sensors to O_2, CH_4, and $H_2O(g)$:

$$[CH_4] = \frac{\left(\frac{R_S}{R_0 P_{O_2}^{\beta}}\right)^{-1/\beta} - 1 - K_{H_2O}[H_2O]}{K_{CH_4}} \quad \textbf{\textit{Clifford equation}} \qquad (4)$$

In this equation R_0 is the sensor resistance in dry air while R_s is the resistance at certain CH_4 and $H_2O(g)$ concentration, P_{O_2} is the relative oxygen partial pressure (being one for air), β is the power law exponent which varies from one sensor to another from 0.25 to 0.55, $[CH_4]$ and $[H_2O]$ are the concentrations of methane and water vapor expressed in volumetric ppm and K_{CH_4} and K_{H_2O} are constants of dimensions ppm^{-1}.

Other attempts to quantify CH_4 via field calibrations[4,5] are exemplified by:

$$[CH_4] = 1.8280 + 0.0288 \left(\frac{R_S}{R_0}\right)_{corr} \quad \textbf{\textit{Eugster \& Kling equation}} \qquad (5)$$

$$[CH_4] = -7.37 + 12.74 \left(\frac{R_S}{R_0}\right)_{corr} \quad \textbf{\textit{Riddick linear equation}} \qquad (6)$$

$$[CH_4] = 1.8 + 0.09\, exp\left(11.669\left(\left(\frac{R_S}{R_0}\right)_{corr} - 0.7083\right)\right) \quad \textbf{\textit{Riddick non-linear equation}} \qquad (7)$$

With:

$$\left(\frac{R_S}{R_0}\right)_{corr} = \left(\frac{R_S}{R_0}(0.024 + 0.072 \cdot rH + 0.0246 \cdot T_a)\right) \qquad (8)$$

Where rH is the relative humidity in % and T_a the air temperature in °C.

In our LCSS, the TGS sensors are connected in series to a resistor ($R_L = 4.7\ k\Omega$), the circuit voltage supplied is 5 V (V_C), and the voltage measured (V_L) varies in relation to the sensing material resistance (R_S). Therefore, to calculate $\left(\frac{R_S}{R_0}\right)_{corr}$ we use[6]:

$$R_S = \left(\frac{V_C}{V_L} - 1\right) \cdot R_L \tag{9}$$

$$\frac{R_S}{R_0} = \frac{\left(\frac{V_C}{V_L} - 1\right)}{\left(\frac{V_C}{V_0} - 1\right)} \tag{10}$$

Where V_0 is the lowest measured sensor output voltage measured during lab calibration at 0 ppm CH_4 with the GMS at 12.5 g·m^{-3} $H_2O(g)$ (35% RH at 33 °C) for the Wetland site, 12.1 g·m^{-3} $H_2O(g)$ (30% RH at 33 °C) for the Sludge screw room and Sludge piles, or at minimum background levels, 11.8 g·m^{-3} $H_2O(g)$ (61% RH at 22 °C) for the Garden at 2.069 ppm CH_4.

We used the equations from former methods to quantify CH_4 using data from various of our data sets from different field sites, and compared the results with our approach, as shown in Table S5 and Figures S8 to S11. For our approach, it must be considered that 20% of the data was used to test the model and that we plot the two-minute means of the UGGA to make it correspond to our trained model. From these results it can be observed that the generalized equations proposed by former methods do not adapt to the particularities that different situations or sensors present, except for the case of Clifford equation, that was elaborated and tested only under controlled laboratory conditions. Therefore, the coefficients from the former methods would need to be adapted for every sensor as our model does, giving new quantification coefficients for each particular set of sensors and field site. For this reason, the former methods

were evaluated also by fitting the equation coefficients to the same data used to train our model (referred to as adjusted coefficients). The coefficients were adjusted, i.e., fitted with our data, by using iterative least squares estimation. To ensure that the comparison between former and current methods are made with comparable data, the former methods were also tested with the two-minute interval data. The R^2 results obtained for the previous methods improved 0.1 in the best case (see Table S6), while the RMSE decreased down to 3.7 ppm for the methods with negative R^2 values and increased up to 2.2 ppm for the rest.

Table S5. Comparisons between published approaches

	**Wetland**		**Sludge screw room**		**Sludge piles**		**Garden**	
	**RMSE (ppm)**	**R^2**	**RMSE (ppm)**	**R^2**	**RMSE (ppm)**	**R^2**	**RMSE (ppm)**	**R^2**
Eugster & Kling	0.2	-0.96	20.4	-1.39	0.9	-0.71	0.1	-15.56
Riddick linear	4.4	-67.92	23.7	-1.81	3.4	-4.77	1.3	-636.42
Riddick nonlinear	7.2	-0.01	20.4	-1.39	0.9	-0.65	0.24	-9.83
Clifford	0.5	-2.20	20.3	-1.37	1.9	-3.17	1.9	$-4.4\cdot10^{3}$
Eugster & Kling adjusted	0.5	0.02	1.7	0.56	0.1	$7.8\cdot10^{-4}$	$5.4\cdot10^{-3}$	0.003
Riddick linear adjusted	0.5	0.02	1.7	0.56	0.1	$7.8\cdot10^{-4}$	$5.4\cdot10^{-3}$	0.003
Riddick nonlinear adjusted	0.5	0.02	11.6	$1.1\cdot10^{-16}$	0.1	0.05	$2.3\cdot10^{-2}$	0.016
Our method (PLSR)	0.1	0.34	5.4	0.91	0.4	0.69	0.32	0.37

Notes: Comparison of root mean squared error and R^2 using different published approaches including coefficients proposed as well as coefficients adjusted to the same error measures obtained for the partial least squares regression method presented in this work for the Wetland, Sludge screw room, sludge piles, and garden field data. Note that R^2 can be negative when the mean value represents the data better than the fit obtained[7].

Table S6. Comparison between approaches for 1- or 2-min averaged data

	Sludge screw room (2-min average)		Sludge screw room (following original descriptions)	
	RMSE (ppm)	R^2	RMSE (ppm)	R^2
Eugster & Kling	18.4	-1.42	20.4	-1.39
Riddick linear	20.0	-1.56	23.7	-1.81
Riddick nonlinear	18.3	-1.42	20.4	-1.39
Clifford	19.0	-1.48	20.3	-1.37
Eugster & Kling adjusted	2.8	0.57	1.7	0.56
Riddick linear adjusted	2.8	0.57	1.7	0.56
Riddick nonlinear adjusted	13.6	0	11.6	$1.1\cdot10^{-16}$
Our method (PLSR)	5.4	0.91	5.4	0.91

Notes: Comparison of root mean squared error and R^2 using 1- or 2-min averaged data for the previous published approaches and the method presented in this work for the Sludge screw room (best results). Note that R^2 can be negative when the slope and/or the intercept terms are affix so that the mean value represents the data better than the fit obtained[7].

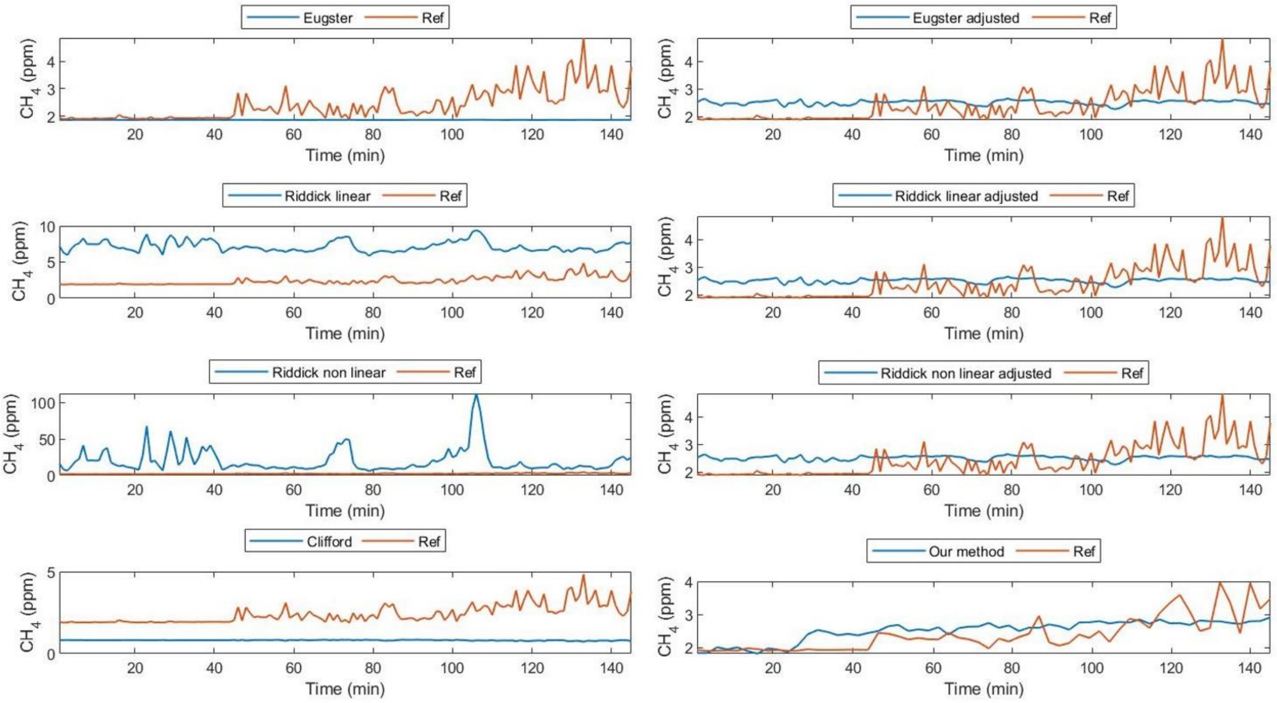

Figure S8. Comparison between methane concentration reported from reference measurements and methane concentrations calculated with previously published methods and the PLSR method presented in this work for the Wetland field site.

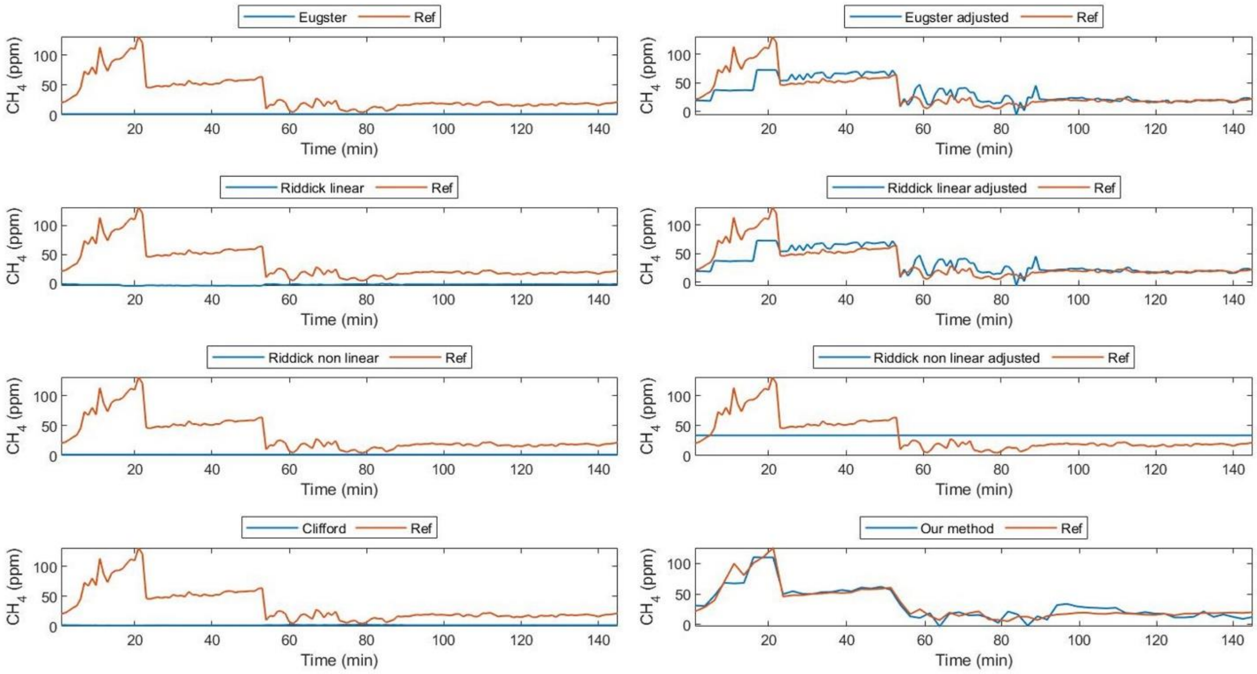

Figure S9. Comparison between methane concentration reported from reference measurements and methane concentrations calculated with previously published methods and the PLSR method presented in this work for the Sludge screw room field site.

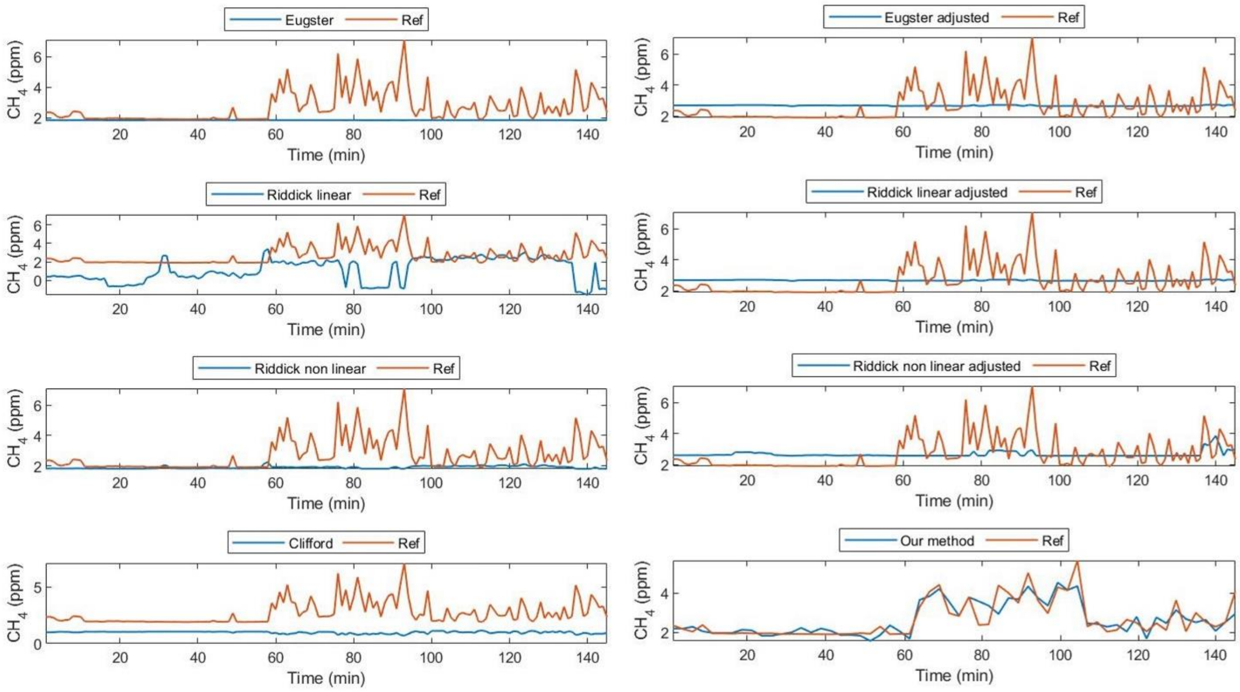

Figure S10. Comparison between methane concentration reported from reference measurements and methane concentrations calculated with previously published methods and the PLSR method presented in this work for the Sludge piles field site.

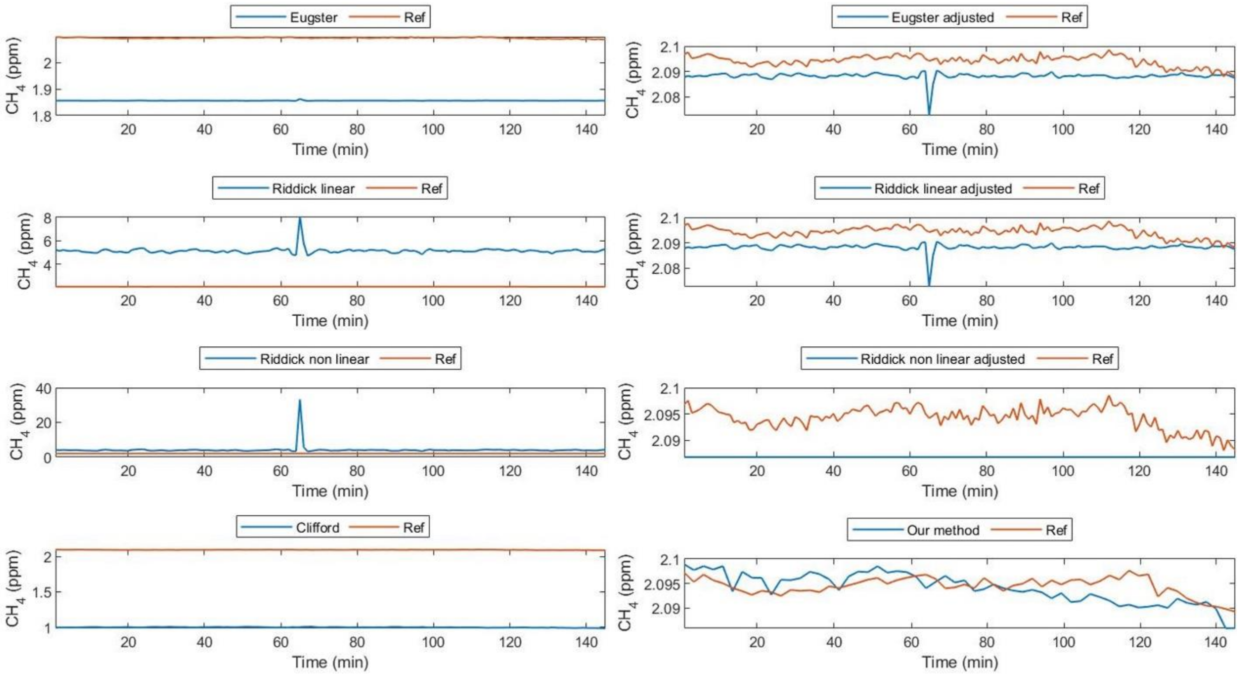

Figure S11. Comparison between methane concentration reported from reference measurements and methane concentrations calculated with previously published methods and the PLSR method presented in this work for the Garden field site.

9. ACCURACY STUDY

To evaluate how R^2 and RMSE depend on the number of points used to train and test the PLSR, we used the case with the largest amount of reference data (Garden). Figure S12 shows the RMSE and R^2 as a function of data points. The R^2 and $RMSE_{train}$ decrease with increasing number of data points, and $RMSE_{test}$ is always lower than $RMSE_{train}$ (always below 100 ppb). The later points out that our model is not over fitted, otherwise $RMSE_{train} < RMSE_{test}$. Distance between R_{train}^2 and R_{test}^2 can help to understand if the model generalizes well, i.e., if the model would predict correct concentrations with unseen data. Larger distance between R_{train}^2 and R_{test}^2 means less ability to generalize. Thus, our model generalizes well in most cases.

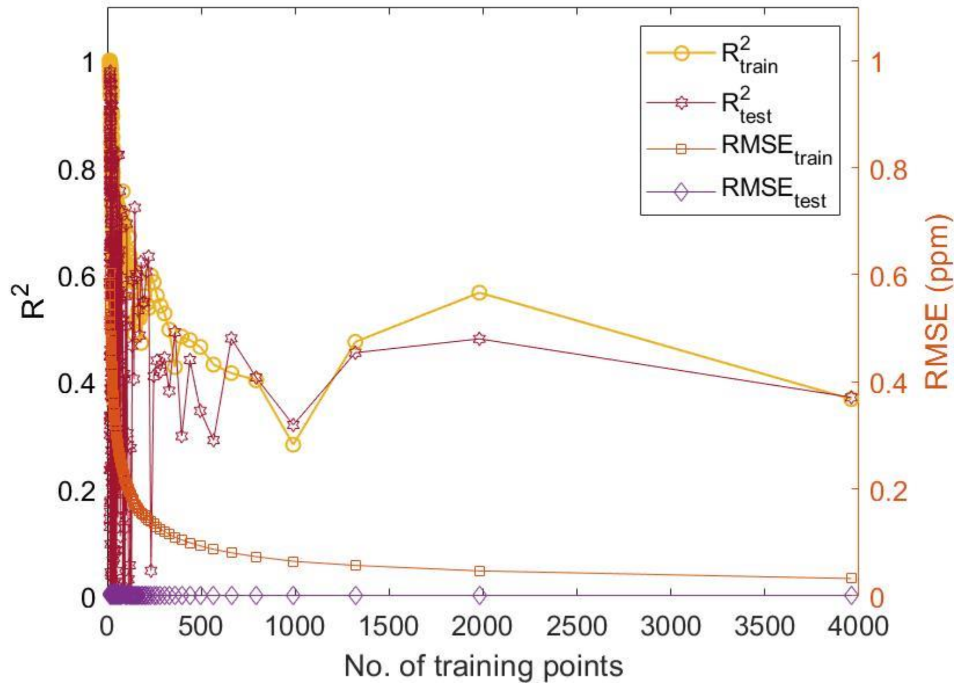

Figure S12. Comparison of R^2 and RMSE as a function of the number of data points used for training and testing the PLSR. Train-test proportion was kept as 80-20% of the whole data set.

```
%% This code is designed by Guillem Domènech-Gil
% When using it cite the manuscript "Electronic nose for improved
% environmental methane monitoring" from 2023

% To use this code you need 3 sensors to measure methane + 1 sensor
to
% measure relative humidity + 1 sensor to measure temperature + 1
sensor to
% measure barometric pressure + reference measurements (in ppm)
% Necessary functions are available at the end of this code

% First let's clean the workspace, graphs, and command window
close all
clear all
clc

%% Upload the data to train the model - MODIFY WITH YOUR DATA

% Type the address where you store the excel file with all the data
in your computer here
% e.g. 'C:\Documents\GasMeasurements\MyFile.xlsx'
Field_data=xlsread('A004_LGR_all_Skogaryd.xlsx');

% Define the columns where you have each sensor and the reference
% measurements, e.g., if the data for Sensor 1 is in column X, then
Sensor1_Field=Field_data(:,X)
Sensor1_Field=Field_data(:,28);  % Type the column with the data
from your sensor 1 here
Sensor2_Field=Field_data(:,30);  % Type the column with the data
from your sensor 2 here
Sensor3_Field=Field_data(:,32);  % Type the column with the data
from your sensor 3 here
RH_Field=Field_data(:,58);      % Type the column with the data from
your relative humidity sensor here
T_Field=Field_data(:,57);       % Type the column with the data from
your temperature sensor here
P_Field=Field_data(:,52);       % Type the column with the data from
your pressure sensor here
UGGA_Field_CH4=Field_data(:,1); % Type the column with the data from
your reference measurement here

% We gather all the data in one matrix to remove rows with missing
values - DO NOT MODIFY
Data_together(:,1)=Sensor1_Field;
Data_together(:,2)=Sensor2_Field;
Data_together(:,3)=Sensor3_Field;
Data_together(:,4)=RH_Field;
Data_together(:,5)=T_Field;
Data_together(:,6)=P_Field;
Data_together(:,7)=UGGA_Field_CH4;

Data_together = rmmissing(Data_together);

Sensor1_Field=Data_together(:,1);
Sensor2_Field=Data_together(:,2);
Sensor3_Field=Data_together(:,3);
```

```
RH_Field=Data_together(:,4);
T_Field=Data_together(:,5);
P_Field=Data_together(:,6);
UGGA_Field_CH4=Data_together(:,7);

% Select the number of point to calculate features. Minimum x=2.
Rememeber
% to always maintain the number of final observations (rows in the
resulting feature
% matrix) at least five times higher than the number of features
(=columns in the
% feature matrix, that will be 18). Recommended, at least 100
observations/rows.
x=2;

%% Upload the data to check how the model performs (Once the
quantification coefficients are calculated, the data set you want to
find methane of) - MODIFY WITH YOUR DATA
% This data set has to be different from the one used for training
and
% testing the model but the sensors used have to be the same ones

% Type the address where you store the excel file with all the data
in your computer here
% e.g. 'C:\Documents\GasMeasurements\MyFile.xlsx'
Field_data4_long=xlsread('C:\Users\guido76\Documents\TEMA\Gas
measurements\2021\Skogaryd
raw\202109_to_20211027_SDcards\A004\A004_alldays_&_LGR.xlsx');

% Define the columns where you have each sensor and the reference
% measurements, e.g., if the data for Sensor 1 is in column X, then
Sensor1_Field=Field_data(:,X)
Sensor1_Field_4_long=Field_data4_long(:,10); % Type the column with
the data from your sensor 1 here
Sensor2_Field_4_long=Field_data4_long(:,12); % Type the column with
the data from your sensor 2 here
Sensor3_Field_4_long=Field_data4_long(:,14); % Type the column with
the data from your sensor 3 here
RH_Field_4_long=Field_data4_long(:,40);      % Type the column with
the data from your relative humidity sensor here
T_Field_4_long=Field_data4_long(:,39);       % Type the column with
the data from your temperature sensor here
P_Field_4_long=Field_data4_long(:,34);       % Type the column with
the data from your pressure sensor here
UGG_Field_4_long=Field_data4_long(:,63)/100000; % Type the column
with the data from your reference measurement here

%% Calculate features - DO NOT MODIFY

[features_mean_value_static_Field]=static_mv(length(Sensor1_Field),S
ensor1_Field,x,1,x);
[features_slope_static_Field]=static_slope(length(Sensor1_Field),Sen
sor1_Field,x,1,x);
[features_fft_static_Field]=static_transform(length(Sensor1_Field),S
ensor1_Field,x,1,x);
```

```
[features_integral_static_Field]=static_integral(length(Sensor1_Fiel
d),Sensor1_Field,x,1,x);
[features_lifting_static_Field]=static_lifting(length(Sensor1_Field)
,Sensor1_Field,x,1,x);

[features2_mean_value_static_Field]=static_mv(length(Sensor2_Field),
Sensor2_Field,x,1,x);
[features2_slope_static_Field]=static_slope(length(Sensor2_Field),Se
nsor2_Field,x,1,x);
[features2_fft_static_Field]=static_transform(length(Sensor2_Field),
Sensor2_Field,x,1,x);
[features2_integral_static_Field]=static_integral(length(Sensor2_Fie
ld),Sensor2_Field,x,1,x);
[features2_lifting_static_Field]=static_lifting(length(Sensor2_Field
),Sensor2_Field,x,1,x);

[features3_mean_value_static_Field]=static_mv(length(Sensor3_Field),
Sensor3_Field,x,1,x);
[features3_slope_static_Field]=static_slope(length(Sensor3_Field),Se
nsor3_Field,x,1,x);
[features3_fft_static_Field]=static_transform(length(Sensor3_Field),
Sensor3_Field,x,1,x);
[features3_integral_static_Field]=static_integral(length(Sensor3_Fie
ld),Sensor3_Field,x,1,x);
[features3_lifting_static_Field]=static_lifting(length(Sensor3_Field
),Sensor3_Field,x,1,x);

[features_RH_Field]=static_mv(length(RH_Field),RH_Field,x,1,x);
[features_T_Field]=static_mv(length(T_Field),T_Field,x,1,x);
[features_P_Field]=static_mv(length(P_Field),P_Field,x,1,x);

Nfeatures1_Field=[features_mean_value_static_Field
features_slope_static_Field features_fft_static_Field
features_integral_static_Field features_lifting_static_Field
features_RH_Field];
Nfeatures2_Field=[features2_mean_value_static_Field
features2_slope_static_Field features2_fft_static_Field
features2_integral_static_Field features2_lifting_static_Field
features_T_Field];
Nfeatures3_Field=[features3_mean_value_static_Field
features3_slope_static_Field features3_fft_static_Field
features3_integral_static_Field features3_lifting_static_Field
features_P_Field];
Nfeatures_Field=[Nfeatures1_Field Nfeatures2_Field
Nfeatures3_Field];

Nfeatures_Field_mv=mean(Nfeatures_Field);
Nfeatures_Field_std=std(Nfeatures_Field);
Nfeatures_Field_standardized=((Nfeatures_Field-
Nfeatures_Field_mv)./Nfeatures_Field_std);

target_Field=static_mv(length(UGGA_Field_CH4),UGGA_Field_CH4,x,1,x);

cv=cvpartition(size(Nfeatures_Field_standardized,1),'Holdout',0.2);
test=cv.test;
```

```
target_train=target_Field(~test,1);
target_test=target_Field(test,1);

train=Nfeatures_Field(~test,:);
test=Nfeatures_Field(test,:);

train_m=mean(train);
train_std=std(train);

target_train_m=mean(target_train);
target_train_std=std(target_train);

train_standardized=((train - train_m)./train_std);
target_train_standardized=((target_train -
target_train_m)./target_train_std);

test_m=mean(test);
test_std=std(test);

target_test_m=mean(target_test);
target_test_std=std(target_test);

test_standardized=((test - test_m)./test_std);
target_test_standardized=((target_test -
target_test_m)./target_test_std);

[XL,YL,~,YS,beta,pctvar,MSE,STATS]=plsregress(train_standardized,
target_train_standardized,size(train_standardized,2), 'CV',10);

features_fitted=[ones(size(train_standardized,1),1)
train_standardized]*beta;
test_fitted=[ones(size(test_standardized,1),1)
test_standardized]*beta;
features_fitted_ppm =
((features_fitted.*target_train_std)+target_train_m);
target_ppm
=((target_train_standardized.*target_train_std)+target_train_m); % Y
test_fitted_ppm=(test_fitted.*target_test_std)+target_test_m;

RMSE_ppm=sqrt(mean((target_train-
features_fitted).^2)/length(target_train_standardized));
RMSE_ppb=RMSE_ppm*1000;

TSS = sum((target_ppm-mean(target_ppm)).^2);
RSS = sum((target_ppm-features_fitted_ppm).^2);
Rsquared = 1 - RSS/TSS;

Sensor1_Field_4_long(isnan(Sensor1_Field_4_long))=0;
Sensor2_Field_4_long(isnan(Sensor2_Field_4_long))=0;
Sensor3_Field_4_long(isnan(Sensor3_Field_4_long))=0;

RH_Field_4_long(isnan(RH_Field_4_long))=0;
T_Field_4_long(isnan(T_Field_4_long))=0;
P_Field_4_long(isnan(P_Field_4_long))=0;
```

```
[features_mean_value_static_Field4_long]=static_mv(length(Sensor1_Fi
eld_4_long),Sensor1_Field_4_long,x,1,x);
[features_slope_static_Field4_long]=static_slope(length(Sensor1_Fiel
d_4_long),Sensor1_Field_4_long,x,1,x);
[features_fft_static_Field4_long]=static_transform(length(Sensor1_Fi
eld_4_long),Sensor1_Field_4_long,x,1,x);
[features_integral_static_Field4_long]=static_integral(length(Sensor
1_Field_4_long),Sensor1_Field_4_long,x,1,x);
[features_lifting_static_Field4_long]=static_lifting(length(Sensor1_
Field_4_long),Sensor1_Field_4_long,x,1,x);

[features2_mean_value_static_Field4_long]=static_mv(length(Sensor2_F
ield_4_long),Sensor2_Field_4_long,x,1,x);
[features2_slope_static_Field4_long]=static_slope(length(Sensor2_Fie
ld_4_long),Sensor2_Field_4_long,x,1,x);
[features2_fft_static_Field4_long]=static_transform(length(Sensor2_F
ield_4_long),Sensor2_Field_4_long,x,1,x);
[features2_integral_static_Field4_long]=static_integral(length(Senso
r2_Field_4_long),Sensor2_Field_4_long,x,1,x);
[features2_lifting_static_Field4_long]=static_lifting(length(Sensor2
_Field_4_long),Sensor2_Field_4_long,x,1,x);

[features3_mean_value_static_Field4_long]=static_mv(length(Sensor3_F
ield_4_long),Sensor3_Field_4_long,x,1,x);
[features3_slope_static_Field4_long]=static_slope(length(Sensor3_Fie
ld_4_long),Sensor3_Field_4_long,x,1,x);
[features3_fft_static_Field4_long]=static_transform(length(Sensor3_F
ield_4_long),Sensor3_Field_4_long,x,1,x);
[features3_integral_static_Field4_long]=static_integral(length(Senso
r3_Field_4_long),Sensor3_Field_4_long,x,1,x);
[features3_lifting_static_Field4_long]=static_lifting(length(Sensor3
_Field_4_long),Sensor3_Field_4_long,x,1,x);

[features_RH_Field4_long]=static_mv(length(RH_Field_4_long),RH_Field
_4_long,x,1,x);
[features_P_Field4_long]=static_mv(length(P_Field_4_long),P_Field_4_
long,x,1,x);
[features_T_Field4_long]=static_mv(length(T_Field_4_long),T_Field_4_
long,x,1,x);

Nfeatures1_Field4_long=[features_mean_value_static_Field4_long
features_slope_static_Field4_long features_fft_static_Field4_long
features_integral_static_Field4_long
features_lifting_static_Field4_long features_RH_Field4_long];
Nfeatures2_Field4_long=[features2_mean_value_static_Field4_long
features2_slope_static_Field4_long features2_fft_static_Field4_long
features2_integral_static_Field4_long
features2_lifting_static_Field4_long features_T_Field4_long];
Nfeatures3_Field4_long=[features3_mean_value_static_Field4_long
features3_slope_static_Field4_long features3_fft_static_Field4_long
features3_integral_static_Field4_long
features3_lifting_static_Field4_long features_P_Field4_long];

Nfeatures_Field4_long=[Nfeatures1_Field4_long Nfeatures2_Field4_long
Nfeatures3_Field4_long];
```

```
Nfeatures_Field4_long =
Nfeatures_Field4_long(all(Nfeatures_Field4_long,2),:);

Nfeatures_Field4_mv_long=mean(Nfeatures_Field4_long);
Nfeatures_Field4_std_long=std(Nfeatures_Field4_long);
Nfeatures_Field4_standardized_long=((Nfeatures_Field4_long-
Nfeatures_Field4_mv_long)./Nfeatures_Field4_std_long);

Nfeatures_Field4_standardized_fitted_long=[ones(size(Nfeatures_Field
4_standardized_long,1),1) Nfeatures_Field4_standardized_long]*beta;
Nfeatures_Field4_standardized_fitted_ppm_long =
((Nfeatures_Field4_standardized_fitted_long.*target_train_std)+targe
t_train_m);

%% plot results - MODIFY IF NECESSARY

xx=[(round(min(target_train))-1):(round(max(target_train))+1)];

figure()
plot(target_train,features_fitted_ppm,'bo')
hold on
plot(target_test,test_fitted_ppm,'c*')
hold on
plot(xx, xx(:)-RMSE_ppm, 'r--')
hold on
plot(xx, xx(:)+RMSE_ppm, 'r--')
hold on
plot(xx,xx,'k-')
ylim([(round(min(target_train))-1) (round(max(target_train))+1)])
xlabel('Reference concentration (ppm)')
ylabel('Predicted concentration (ppm)')
legend('Training data','Test
data','RMSE','FontSize',14,'Location','Northwest')
annotation('textbox', [0.15, 0.62, 0.1, 0.1],'String', strcat('R^2 =
',num2str(Rsquared)),'FontSize',12,LineStyle='none');
annotation('textbox', [0.15, 0.54, 0.1, 0.1],'String', strcat('RMSE
=  ', num2str(RMSE_ppb),' ppb'),'FontSize',12,LineStyle='none');
legend('boxoff')

% Printed training data versus reference measurement as temporal
evolution

% Change xlabel, xticks, and xticklabels according to the timing of
your data
figure()
plot(target_train,'r*-',LineWidth=0.5)
hold on
plot(features_fitted_ppm,'bo-',LineWidth=0.5)
xlabel('Time (X)','Fontsize',12')
ylabel('CH_4 concentration (ppm)')
legend('PLSR trained data','Reference signal','Fontsize',12)

xticks([43920/x 87840/x  131760/x  175680/x  219600/x  263520/x
307440/x 351360/x])
xticklabels({'Month 1','Month 2','Month 3','Month 4','Month
5','Month 6','Month 7','Month 8'})
```

```
% Printed test data versus reference measurement as temporal
evolution

% Change xlabel, xticks, and xticklabels according to the timing of
your data
figure()
plot(target_test,'ro-',LineWidth=1)
hold on
plot(test_fitted_ppm,'c*-',LineWidth=1)

xlabel('Time (h)','Fontsize',12')
ylabel('CH_4 concentration (ppm)')
legend('UGGA test data','PLSR test data','Fontsize',12)
xticks([180/x 360/x 540/x 720/x 900/x 1080/x 1260/x 1440/x 1620/x
1800/x 1980/x 2160/x 2340/x 2520/x 2880/x ])
xticklabels({'3','6','9','12','15','18','21','24','27','30','33','36
','39','42','45'})

% Plot temporal evolution of the methane concentration calculated

% Change xlabel, xticks, and xticklabels according to the timing of
your data
figure()
plot(Nfeatures_Field4_standardized_fitted_ppm_long,'bo-
',LineWidth=0.5)
hold on
plot(UGG_Field_4_long,'r*-')
xlabel('Time (d)','Fontsize',12')
ylabel('CH_4 concentration (ppm)')
legend('Test PLSR prediction','UGGA test signal','Fontsize',12)
xticks([1440/x 2880/x 4320/x 5760/x 7200/x 8640/x 10080/x 11520/x
12960/x 14400/x 15840/x 17280/x 18720/x 20160/x 21600/x 23040/x
24480/x 25920/x 27360/x 28800/x 30240/x])
xticklabels({'1','2','3','4','5','6','7','8','9','10','11','12','13'
,'14','15','16','17','18','19','20','21','22','23','24','25'})
```

```
%% This code is designed by Guillem Domènech-Gil
% When using it cite the manuscript "Electronic nose for improved
% environmental methane monitoring" from 2023

% Extract mean value as a feature

function
[mean_value_features_static]=static_mv(signal_length,sensor_signal,x
,y,z)

% signal_lenght = number of data points of the sensor signal
% sensor_signal = data acquired of the sensor signal during
calibration
% x = lenght of each step
% y = first point for mean value calculation
% z = last point for mean value calculation

    for i=1:signal_length/x

mean_value_features_static(i,1)=mean(sensor_signal((y:z)+x*(i-
1),1));
    end

end
```

```
%% This code is designed by Guillem Domènech-Gil
% When using it cite the manuscript "Electronic nose for improved
% environmental methane monitoring" from 2023

% Extract slope as a feature

function
[slope_features_static]=static_slope(signal_length,sensor_signal,x,y
,z)

% signal_lenght = number of data points of the sensor signal
% sensor_signal = data acquired of the sensor signal during
calibration
% x = lenght of each step
% y = first point for polyfit calculation
% z = last point for polyfit calculation

a=[1:z-y+1];
a=a';
for i=1:signal_length/x
    polyn((1:2)+2*(i-1),1)=polyfit(a,sensor_signal((y:z)+x*(i-
1),1),1);
end
%
for i=1:signal_length/x
        slope_features_static(i,1)=polyn(i+(i-1),1);
end

end
```

```
%% This code is designed by Guillem Domènech-Gil
% When using it cite the manuscript "Electronic nose for improved
% environmental methane monitoring" from 2023

% Extract FFT as a feature

function
[fft_features_static]=static_transform(signal_length,sensor_signal,x
,y,z)


% signal_lenght = number of data points of the sensor signal
% sensor_signal = data acquired of the sensor signal during
calibration
% x = lenght of each step
% y = first point for fast fourier transform calculation
% z = last point for fast fourier transform calculation

for i=1:signal_length/x

fft_features_static(i,1)=log(abs(fft2(mean(sensor_signal((y:z)+x*(i-
1),1)))));
end
%abs(sec(mean(fft
end
```

```
%% This code is designed by Guillem Domènech-Gil
% When using it cite the manuscript "Electronic nose for improved
% environmental methane monitoring" from 2023

% signal_lenght = number of data points of the sensor signal
% sensor_signal = data acquired of the sensor signal during
calibration
% target = reference data acquired of the sensor signal during
calibration
% x is the amount used as test, between 0 and 1. If you want to use
30% of the data to test your model, use x=0.3
% y is the number of folds used for the cross validation


function [target_train,features_fitted_ppm,target_test,
test_fitted_ppm, RMSE_ppm,
RMSE_ppb,R2]=cv_plsr_errors(signal_length,sensor_signal,target,x,y)

cv=cvpartition(signal_length,'Holdout',x);
test=cv.test;

target_train=target(~test,1);
target_test=target(test,1);

train=sensor_signal(~test,:);
test=sensor_signal(test,:);

train_m=mean(train);
train_std=std(train);

target_train_m=mean(target_train);
target_train_std=std(target_train);

tr_stand=((train - train_m)./train_std);
targ_tr_stand=((target_train - target_train_m)./target_train_std);

test_m=mean(test);
test_std=std(test);

target_test_m=mean(target_test);
target_test_std=std(target_test);

te_stand=((test - test_m)./test_std);
targ_te_stand=((target_test - target_test_m)./target_test_std);

[XL,YL,~,YS,beta,pctvar,MSE,STATS]=plsregress(tr_stand,
targ_tr_stand, size(tr_stand,2), 'CV',y);


features_fitted=[ones(size(tr_stand,1),1) tr_stand]*beta;
test_fitted=[ones(size(te_stand,1),1) te_stand]*beta;
features_fitted_ppm =
((features_fitted.*target_train_std)+target_train_m);
target_ppm =((targ_tr_stand.*target_train_std)+target_train_m);
test_fitted_ppm=(test_fitted.*target_test_std)+target_test_m;
```

```
RMSE_ppm=sqrt(mean((target_train-
features_fitted).^2)/length(targ_tr_stand));
RMSE_ppb=RMSE_ppm*1000;

TSS = sum((target_ppm-mean(target_ppm)).^2);
RSS = sum((target_ppm-features_fitted_ppm).^2);
R2 = 1 - RSS/TSS;

end
```

REFERENCES

[1] Manolakis, D., Ingle, V. Chapter 7 and 8. In: *Applied Digital signal processing*, Cambridge University Press, 2011, pp 353-474.

[2] Moseley, P.T. Solid state gas sensors. *Meas. Sci. Technol.* **1997**, 8, 223. https://doi.org/10.1088/0957-0233/8/3/003.

[3] Barsan, N., Weimar, U. Conduction Model of Metal Oxide Gas Sensors. *Journal of Electroceramics* **2001**, 7, 143- 167. http://dx.doi.org/10.1023/A:1014405811371.

[4] Riddick, S. N, Mauzerall D. L, Celia, M. Allen, G., Pitt, J., Kang, M. and Riddick, J. C. The calibration and deployment of a low-cost methane sensor. *Atmos. Environ.* **2020**, 230, 117440. http://dx.doi.org/10.1016/j.atmosenv.2020.117440.

[5] Eugster, W.; Laundre, J.; Eugster, J.; Kling, G.W.; Long-term reliability of the Figaro TGS 2600 solid-state methane sensor under low-Arctic conditions at Toolik Lake, Alaska. *Atmos. Meas. Tech.* **2020**, 13, 2681-2695. https://doi.org/10.5194/amt-13-2681-2020.

[6] Bastviken, D.; Nygren, J.; Schenk, J.; Massana, R.P.; Duc, N.T. Technical note: Facilitating the use of low-cost methane (CH_4) sensors in flux chambers – calibration, data processing, and an open-source make-it-yourself logger. *Biogeosciences* **2020**, 17, 3659-3667. https://doi.org/10.5194/bg-17-3659-2020.

[7] Chicco, D.; Warrens, J.M.; Jurman, G. The coefficient of determination R-squared is more informative than SMAPE, MAE, MAPE, MSE and RMSE in regression analysis evaluation. *PeerJ Comput. Sci.* **2021**, 7, e623. https://doi.org/10.7717/peerj-cs.623.
